# Supplementary material for: Investigation of positive mental health levels among faculty of health sciences students at a rural university in South Africa
Source: PLOS Glob Public Health. 2024 Jul 29;4(7):e0002855. doi: 10.1371/journal.pgph.0002855 (PMC11285916; doi:10.1371/journal.pgph.0002855)
Supplement: S1 Data — (DOCX) [file pgph.0002855.s002.docx]

**Frequencies**

|  | | Gender | Ethnicity | Age | LevelEdu | Bursary | Relaffil | HouholdInc |
| --- | --- | --- | --- | --- | --- | --- | --- | --- |
| N | Valid | 354 | 354 | 354 | 354 | 354 | 354 | 354 |
|  | Missing | 0 | 0 | 0 | 0 | 0 | 0 | 0 |

| FamRes | Relstatus | HisPsyIll | MedTrePsyIll | Living |
| --- | --- | --- | --- | --- |
| 354 | 354 | 354 | 354 | 354 |
| 0 | 0 | 0 | 0 | 0 |

**Frequency Table**

| **Gender** | | | | | |
| --- | --- | --- | --- | --- | --- |
|  | | Frequency | Percent | Valid Percent | Cumulative Percent |
| Valid | Male | 142 | 40.1 | 40.1 | 40.1 |
|  | Female | 212 | 59.9 | 59.9 | 100.0 |
|  | Total | 354 | 100.0 | 100.0 |  |

| **Race** | | | | | |
| --- | --- | --- | --- | --- | --- |
|  | | Frequency | Percent | Valid Percent | Cumulative Percent |
| Valid | Black | 351 | 99.2 | 99.2 | 99.2 |
|  | White | 3 | .8 | .8 | 100.0 |
|  | Total | 354 | 100.0 | 100.0 |  |

| **Age** | | | | | |
| --- | --- | --- | --- | --- | --- |
|  | | Frequency | Percent | Valid Percent | Cumulative Percent |
| Valid | 18-19 Years | 96 | 27.1 | 27.1 | 27.1 |
|  | 20-21 Years | 131 | 37.0 | 37.0 | 64.1 |
|  | 22-23 Years | 84 | 23.7 | 23.7 | 87.9 |
|  | >23 Years | 43 | 12.1 | 12.1 | 100.0 |
|  | Total | 354 | 100.0 | 100.0 |  |

| **Field of study** | | | | | |
| --- | --- | --- | --- | --- | --- |
|  | | Frequency | Percent | Valid Percent | Cumulative Percent |
| Valid | Pharmacy | 61 | 17.2 | 17.2 | 17.2 |
|  | Optometry | 40 | 11.3 | 11.3 | 28.5 |
|  | Nursing | 60 | 16.9 | 16.9 | 45.5 |
|  | HND | 49 | 13.8 | 13.8 | 59.3 |
|  | Medicine | 92 | 26.0 | 26.0 | 85.3 |
|  | Medical Sciences | 52 | 14.7 | 14.7 | 100.0 |
|  | Total | 354 | 100.0 | 100.0 |  |

| **Bursary** | | | | | |
| --- | --- | --- | --- | --- | --- |
|  | | Frequency | Percent | Valid Percent | Cumulative Percent |
| Valid | Yes | 278 | 78.5 | 78.5 | 78.5 |
|  | No | 76 | 21.5 | 21.5 | 100.0 |
|  | Total | 354 | 100.0 | 100.0 |  |

| **Religious affiliation** | | | | | |
| --- | --- | --- | --- | --- | --- |
|  | | Frequency | Percent | Valid Percent | Cumulative Percent |
| Valid | Christianity | 245 | 69.2 | 69.2 | 69.2 |
|  | Other | 109 | 30.8 | 30.8 | 100.0 |
|  | Total | 354 | 100.0 | 100.0 |  |

| **Household income** | | | | | |
| --- | --- | --- | --- | --- | --- |
|  | | Frequency | Percent | Valid Percent | Cumulative Percent |
| Valid | R0-R5000 | 103 | 29.1 | 29.1 | 29.1 |
|  | R5001-R10000 | 79 | 22.3 | 22.3 | 51.4 |
|  | R10001-R20000 | 76 | 21.5 | 21.5 | 72.9 |
|  | >R20000 | 96 | 27.1 | 27.1 | 100.0 |
|  | Total | 354 | 100.0 | 100.0 |  |

| **Family residence** | | | | | |
| --- | --- | --- | --- | --- | --- |
|  | | Frequency | Percent | Valid Percent | Cumulative Percent |
| Valid | Rural | 275 | 77.7 | 77.7 | 77.7 |
|  | Urban | 79 | 22.3 | 22.3 | 100.0 |
|  | Total | 354 | 100.0 | 100.0 |  |

| **Relationship status** | | | | | |
| --- | --- | --- | --- | --- | --- |
|  | | Frequency | Percent | Valid Percent | Cumulative Percent |
| Valid | Single | 255 | 72.0 | 72.0 | 72.0 |
|  | In Relationship/Married | 99 | 28.0 | 28.0 | 100.0 |
|  | Total | 354 | 100.0 | 100.0 |  |

| **History of psychiatric illness** | | | | | |
| --- | --- | --- | --- | --- | --- |
|  | | Frequency | Percent | Valid Percent | Cumulative Percent |
| Valid | Yes | 13 | 3.7 | 3.7 | 3.7 |
|  | No | 341 | 96.3 | 96.3 | 100.0 |
|  | Total | 354 | 100.0 | 100.0 |  |

| **Received treatment for psychiatric illness** | | | | | |
| --- | --- | --- | --- | --- | --- |
|  | | Frequency | Percent | Valid Percent | Cumulative Percent |
| Valid | Yes | 14 | 4.0 | 4.0 | 4.0 |
|  | No | 340 | 96.0 | 96.0 | 100.0 |
|  | Total | 354 | 100.0 | 100.0 |  |

| **Current living status** | | | | | |
| --- | --- | --- | --- | --- | --- |
|  | | Frequency | Percent | Valid Percent | Cumulative Percent |
| Valid | UNIRES | 264 | 74.6 | 74.6 | 74.6 |
|  | PRIV/LIVOTHER | 82 | 23.2 | 23.2 | 97.7 |
|  | LIVPARENT | 8 | 2.3 | 2.3 | 100.0 |
|  | Total | 354 | 100.0 | 100.0 |  |

**T-Test**

| **Group Statistics** | | | | | |
| --- | --- | --- | --- | --- | --- |
|  | Gender | N | Mean | Std. Deviation | Std. Error Mean |
| TPMH | Male | 142 | 4.499 | .6764 | .0568 |
|  | Female | 212 | 4.354 | .6101 | .0419 |
| GC | Male | 142 | 4.286 | .9064 | .0761 |
|  | Female | 212 | 3.875 | 1.0264 | .0705 |
| ES | Male | 142 | 4.363 | 1.1066 | .0929 |
|  | Female | 212 | 4.512 | 1.2473 | .0857 |
| SP | Male | 142 | 4.725 | 1.0267 | .0862 |
|  | Female | 212 | 5.012 | .9536 | .0655 |
| IS | Male | 142 | 4.332 | .8647 | .0726 |
|  | Female | 212 | 4.198 | .8341 | .0573 |
| PGA | Male | 142 | 4.780 | .9571 | .0803 |
|  | Female | 212 | 4.499 | .8201 | .0563 |
| GEF | Male | 142 | 4.547 | 1.0545 | .0885 |
|  | Female | 212 | 4.100 | 1.1500 | .0790 |

| **Independent Samples Test** | | | | | | | | | | | |
| --- | --- | --- | --- | --- | --- | --- | --- | --- | --- | --- | --- |
|  | | Levene's Test for Equality of Variances | | t-test for Equality of Means | | | | | | | |
|  |  | F | Sig. | t | df | Significance | | Mean Difference | Std. Error Difference | 95% Confidence Interval of the Difference | |
|  |  |  |  |  |  | One-Sided p | Two-Sided p |  |  | Lower | Upper |
| TPMH | Equal variances assumed | 1.438 | .231 | 2.098 | 352 | .018 | .037 | .1451 | .0691 | .0091 | .2810 |
|  | Equal variances not assumed |  |  | 2.056 | 280.801 | .020 | .041 | .1451 | .0706 | .0062 | .2839 |
| GC | Equal variances assumed | 3.464 | .064 | 3.871 | 352 | <.001 | <.001 | .4114 | .1063 | .2024 | .6204 |
|  | Equal variances not assumed |  |  | 3.967 | 326.359 | <.001 | <.001 | .4114 | .1037 | .2074 | .6154 |
| ES | Equal variances assumed | 1.991 | .159 | -1.151 | 352 | .125 | .251 | -.1489 | .1294 | -.4033 | .1055 |
|  | Equal variances not assumed |  |  | -1.178 | 325.557 | .120 | .239 | -.1489 | .1263 | -.3974 | .0997 |
| SP | Equal variances assumed | 3.855 | .050 | -2.686 | 352 | .004 | .008 | -.2864 | .1067 | -.4962 | -.0767 |
|  | Equal variances not assumed |  |  | -2.647 | 286.986 | .004 | .009 | -.2864 | .1082 | -.4995 | -.0734 |
| IS | Equal variances assumed | 1.988 | .159 | 1.460 | 352 | .073 | .145 | .1340 | .0918 | -.0465 | .3146 |
|  | Equal variances not assumed |  |  | 1.450 | 294.954 | .074 | .148 | .1340 | .0925 | -.0479 | .3160 |
| PGA | Equal variances assumed | 2.741 | .099 | 2.955 | 352 | .002 | .003 | .2812 | .0952 | .0941 | .4684 |
|  | Equal variances not assumed |  |  | 2.867 | 270.130 | .002 | .004 | .2812 | .0981 | .0881 | .4744 |
| GEF | Equal variances assumed | .758 | .385 | 3.706 | 352 | <.001 | <.001 | .4472 | .1207 | .2099 | .6845 |
|  | Equal variances not assumed |  |  | 3.770 | 319.590 | <.001 | <.001 | .4472 | .1186 | .2138 | .6805 |

**T-Test**

| **Group Statistics** | | | | | |
| --- | --- | --- | --- | --- | --- |
|  | Race | N | Mean | Std. Deviation | Std. Error Mean |
| TPMH | Black | 351 | 4.408 | .6405 | .0342 |
|  | White | 3 | 4.967 | .4041 | .2333 |
| GC | Black | 351 | 4.036 | 1.0027 | .0535 |
|  | White | 3 | 4.433 | .2887 | .1667 |
| ES | Black | 351 | 4.440 | 1.1913 | .0636 |
|  | White | 3 | 5.867 | .1528 | .0882 |
| SP | Black | 351 | 4.898 | .9899 | .0528 |
|  | White | 3 | 4.733 | 1.4844 | .8570 |
| IS | Black | 351 | 4.246 | .8463 | .0452 |
|  | White | 3 | 4.900 | .9539 | .5508 |
| PGA | Black | 351 | 4.605 | .8865 | .0473 |
|  | White | 3 | 5.400 | .6557 | .3786 |
| GEF | Black | 351 | 4.279 | 1.1368 | .0607 |
|  | White | 3 | 4.367 | .5132 | .2963 |

| **Independent Samples Test** | | | | | | | | | | | |
| --- | --- | --- | --- | --- | --- | --- | --- | --- | --- | --- | --- |
|  | | Levene's Test for Equality of Variances | | t-test for Equality of Means | | | | | | | |
|  |  | F | Sig. | t | df | Significance | | Mean Difference | Std. Error Difference | 95% Confidence Interval of the Difference | |
|  |  |  |  |  |  | One-Sided p | Two-Sided p |  |  | Lower | Upper |
| TPMH | Equal variances assumed | .912 | .340 | -1.508 | 352 | .066 | .132 | -.5590 | .3707 | -1.2881 | .1701 |
|  | Equal variances not assumed |  |  | -2.370 | 2.087 | .068 | .136 | -.5590 | .2358 | -1.5343 | .4163 |
| GC | Equal variances assumed | 2.923 | .088 | -.685 | 352 | .247 | .494 | -.3972 | .5799 | -1.5376 | .7433 |
|  | Equal variances not assumed |  |  | -2.269 | 2.434 | .064 | .128 | -.3972 | .1750 | -1.0353 | .2410 |
| ES | Equal variances assumed | 4.892 | .028 | -2.071 | 352 | .020 | .039 | -1.4262 | .6888 | -2.7809 | -.0715 |
|  | Equal variances not assumed |  |  | -13.118 | 4.613 | <.001 | <.001 | -1.4262 | .1087 | -1.7129 | -1.1395 |
| SP | Equal variances assumed | .773 | .380 | .286 | 352 | .387 | .775 | .1650 | .5760 | -.9679 | 1.2978 |
|  | Equal variances not assumed |  |  | .192 | 2.015 | .433 | .865 | .1650 | .8586 | -3.5028 | 3.8327 |
| IS | Equal variances assumed | .143 | .706 | -1.332 | 352 | .092 | .184 | -.6541 | .4910 | -1.6199 | .3116 |
|  | Equal variances not assumed |  |  | -1.184 | 2.027 | .178 | .357 | -.6541 | .5526 | -3.0017 | 1.6935 |
| PGA | Equal variances assumed | .723 | .396 | -1.548 | 352 | .061 | .122 | -.7949 | .5134 | -1.8045 | .2148 |
|  | Equal variances not assumed |  |  | -2.083 | 2.063 | .084 | .169 | -.7949 | .3815 | -2.3894 | .7997 |
| GEF | Equal variances assumed | 1.934 | .165 | -.134 | 352 | .447 | .894 | -.0880 | .6576 | -1.3814 | 1.2053 |
|  | Equal variances not assumed |  |  | -.291 | 2.171 | .398 | .796 | -.0880 | .3024 | -1.2957 | 1.1196 |

**T-Test**

| **Group Statistics** | | | | | |
| --- | --- | --- | --- | --- | --- |
|  | Bursary | N | Mean | Std. Deviation | Std. Error Mean |
| TPMH | Yes | 278 | 4.423 | .6509 | .0390 |
|  | No | 76 | 4.375 | .6036 | .0692 |
| GC | Yes | 278 | 4.051 | 1.0025 | .0601 |
|  | No | 76 | 3.999 | .9932 | .1139 |
| ES | Yes | 278 | 4.471 | 1.2254 | .0735 |
|  | No | 76 | 4.384 | 1.0733 | .1231 |
| SP | Yes | 278 | 4.911 | .9777 | .0586 |
|  | No | 76 | 4.846 | 1.0485 | .1203 |
| IS | Yes | 278 | 4.255 | .8655 | .0519 |
|  | No | 76 | 4.239 | .7852 | .0901 |
| PGA | Yes | 278 | 4.618 | .8805 | .0528 |
|  | No | 76 | 4.588 | .9164 | .1051 |
| GEF | Yes | 278 | 4.299 | 1.1259 | .0675 |
|  | No | 76 | 4.209 | 1.1619 | .1333 |

| **Independent Samples Test** | | | | | | | | | | | | |
| --- | --- | --- | --- | --- | --- | --- | --- | --- | --- | --- | --- | --- |
|  | | Levene's Test for Equality of Variances | | t-test for Equality of Means | | | | | | | | |
|  |  | F | Sig. | t | df | Significance | | Mean Difference | Std. Error Difference | 95% Confidence Interval of the Difference | |  |
|  |  |  |  |  |  | One-Sided p | Two-Sided p |  |  | Lower | Upper |  |
| TPMH | Equal variances assumed | .132 | .717 | .574 | 352 | .283 | .566 | .0477 | .0830 | -.1156 | .2109 |  |
|  | Equal variances not assumed |  |  | .600 | 126.806 | .275 | .550 | .0477 | .0795 | -.1096 | .2049 |  |
| GC | Equal variances assumed | .121 | .728 | .402 | 352 | .344 | .688 | .0520 | .1295 | -.2027 | .3067 |  |
|  | Equal variances not assumed |  |  | .404 | 120.069 | .343 | .687 | .0520 | .1288 | -.2030 | .3071 |  |
| ES | Equal variances assumed | 1.584 | .209 | .563 | 352 | .287 | .574 | .0870 | .1546 | -.2171 | .3911 |  |
|  | Equal variances not assumed |  |  | .607 | 133.392 | .272 | .545 | .0870 | .1434 | -.1966 | .3706 |  |
| SP | Equal variances assumed | .096 | .757 | .504 | 352 | .307 | .615 | .0647 | .1286 | -.1881 | .3176 |  |
|  | Equal variances not assumed |  |  | .484 | 113.162 | .315 | .629 | .0647 | .1338 | -.2003 | .3298 |  |
| IS | Equal variances assumed | .053 | .818 | .138 | 352 | .445 | .890 | .0152 | .1099 | -.2009 | .2313 |  |
|  | Equal variances not assumed |  |  | .146 | 129.234 | .442 | .884 | .0152 | .1040 | -.1905 | .2209 |  |
| PGA | Equal variances assumed | .819 | .366 | .263 | 352 | .397 | .793 | .0302 | .1150 | -.1960 | .2563 |  |
|  | Equal variances not assumed |  |  | .257 | 115.648 | .399 | .798 | .0302 | .1176 | -.2028 | .2632 |  |
| GEF | Equal variances assumed | .046 | .831 | .609 | 352 | .271 | .543 | .0894 | .1467 | -.1992 | .3779 |  |
|  | Equal variances not assumed |  |  | .598 | 116.372 | .275 | .551 | .0894 | .1494 | -.2066 | .3853 |  |

**T-Test**

| **Group Statistics** | | | | | |
| --- | --- | --- | --- | --- | --- |
|  | Religious affiliation | N | Mean | Std. Deviation | Std. Error Mean |
| TPMH | Christianity | 245 | 4.433 | .6383 | .0408 |
|  | Other | 109 | 4.366 | .6460 | .0619 |
| GC | Christianity | 245 | 4.044 | .9883 | .0631 |
|  | Other | 109 | 4.029 | 1.0283 | .0985 |
| ES | Christianity | 245 | 4.462 | 1.1891 | .0760 |
|  | Other | 109 | 4.431 | 1.2087 | .1158 |
| SP | Christianity | 245 | 4.989 | .9430 | .0602 |
|  | Other | 109 | 4.690 | 1.0704 | .1025 |
| IS | Christianity | 245 | 4.282 | .8728 | .0558 |
|  | Other | 109 | 4.183 | .7884 | .0755 |
| PGA | Christianity | 245 | 4.610 | .8725 | .0557 |
|  | Other | 109 | 4.616 | .9232 | .0884 |
| GEF | Christianity | 245 | 4.270 | 1.1464 | .0732 |
|  | Other | 109 | 4.301 | 1.1058 | .1059 |

| **Independent Samples Test** | | | | | | | | | | | |
| --- | --- | --- | --- | --- | --- | --- | --- | --- | --- | --- | --- |
|  | | Levene's Test for Equality of Variances | | t-test for Equality of Means | | | | | | | |
|  |  | F | Sig. | t | df | Significance | | Mean Difference | Std. Error Difference | 95% Confidence Interval of the Difference | |
|  |  |  |  |  |  | One-Sided p | Two-Sided p |  |  | Lower | Upper |
| TPMH | Equal variances assumed | .015 | .902 | .908 | 352 | .182 | .364 | .0670 | .0738 | -.0781 | .2121 |
|  | Equal variances not assumed |  |  | .904 | 205.093 | .183 | .367 | .0670 | .0741 | -.0791 | .2131 |
| GC | Equal variances assumed | .472 | .493 | .128 | 352 | .449 | .898 | .0147 | .1152 | -.2119 | .2413 |
|  | Equal variances not assumed |  |  | .126 | 200.040 | .450 | .900 | .0147 | .1170 | -.2160 | .2454 |
| ES | Equal variances assumed | .117 | .733 | .224 | 352 | .411 | .823 | .0308 | .1376 | -.2398 | .3015 |
|  | Equal variances not assumed |  |  | .223 | 204.265 | .412 | .824 | .0308 | .1385 | -.2422 | .3039 |
| SP | Equal variances assumed | 1.027 | .311 | 2.640 | 352 | .004 | .009 | .2991 | .1133 | .0763 | .5219 |
|  | Equal variances not assumed |  |  | 2.515 | 185.666 | .006 | .013 | .2991 | .1189 | .0645 | .5337 |
| IS | Equal variances assumed | .034 | .853 | 1.005 | 352 | .158 | .315 | .0981 | .0976 | -.0938 | .2901 |
|  | Equal variances not assumed |  |  | 1.046 | 227.901 | .148 | .297 | .0981 | .0939 | -.0868 | .2831 |
| PGA | Equal variances assumed | .823 | .365 | -.053 | 352 | .479 | .958 | -.0054 | .1023 | -.2066 | .1958 |
|  | Equal variances not assumed |  |  | -.052 | 197.123 | .479 | .959 | -.0054 | .1045 | -.2115 | .2007 |
| GEF | Equal variances assumed | .057 | .812 | -.238 | 352 | .406 | .812 | -.0311 | .1306 | -.2879 | .2257 |
|  | Equal variances not assumed |  |  | -.242 | 214.292 | .405 | .809 | -.0311 | .1288 | -.2850 | .2227 |

**T-Test**

| **Group Statistics** | | | | | |
| --- | --- | --- | --- | --- | --- |
|  | Family Residence | N | Mean | Std. Deviation | Std. Error Mean |
| TPMH | Rural | 275 | 4.412 | .6546 | .0395 |
|  | Urban | 79 | 4.413 | .5930 | .0667 |
| GC | Rural | 275 | 4.025 | 1.0090 | .0608 |
|  | Urban | 79 | 4.090 | .9696 | .1091 |
| ES | Rural | 275 | 4.426 | 1.2241 | .0738 |
|  | Urban | 79 | 4.544 | 1.0825 | .1218 |
| SP | Rural | 275 | 4.922 | .9604 | .0579 |
|  | Urban | 79 | 4.809 | 1.0976 | .1235 |
| IS | Rural | 275 | 4.276 | .8748 | .0528 |
|  | Urban | 79 | 4.167 | .7455 | .0839 |
| PGA | Rural | 275 | 4.616 | .8879 | .0535 |
|  | Urban | 79 | 4.596 | .8899 | .1001 |
| GEF | Rural | 275 | 4.267 | 1.1412 | .0688 |
|  | Urban | 79 | 4.323 | 1.1084 | .1247 |

| **Independent Samples Test** | | | | | | | | | | | |
| --- | --- | --- | --- | --- | --- | --- | --- | --- | --- | --- | --- |
|  | | Levene's Test for Equality of Variances | | t-test for Equality of Means | | | | | | | |
|  |  | F | Sig. | t | df | Significance | | Mean Difference | Std. Error Difference | 95% Confidence Interval of the Difference | |
|  |  |  |  |  |  | One-Sided p | Two-Sided p |  |  | Lower | Upper |
| TPMH | Equal variances assumed | .947 | .331 | -.004 | 352 | .499 | .997 | -.0003 | .0819 | -.1613 | .1607 |
|  | Equal variances not assumed |  |  | -.004 | 137.372 | .498 | .997 | -.0003 | .0775 | -.1536 | .1530 |
| GC | Equal variances assumed | .305 | .581 | -.507 | 352 | .306 | .612 | -.0648 | .1277 | -.3159 | .1864 |
|  | Equal variances not assumed |  |  | -.519 | 130.479 | .302 | .605 | -.0648 | .1249 | -.3119 | .1823 |
| ES | Equal variances assumed | 2.479 | .116 | -.775 | 352 | .219 | .439 | -.1181 | .1524 | -.4179 | .1817 |
|  | Equal variances not assumed |  |  | -.829 | 140.441 | .204 | .408 | -.1181 | .1424 | -.3997 | .1634 |
| SP | Equal variances assumed | 1.508 | .220 | .895 | 352 | .186 | .372 | .1133 | .1267 | -.1358 | .3625 |
|  | Equal variances not assumed |  |  | .831 | 114.507 | .204 | .408 | .1133 | .1364 | -.1569 | .3835 |
| IS | Equal variances assumed | .030 | .862 | 1.003 | 352 | .158 | .317 | .1085 | .1082 | -.1043 | .3214 |
|  | Equal variances not assumed |  |  | 1.095 | 145.421 | .138 | .275 | .1085 | .0991 | -.0873 | .3044 |
| PGA | Equal variances assumed | .127 | .722 | .178 | 352 | .429 | .859 | .0202 | .1134 | -.2029 | .2432 |
|  | Equal variances not assumed |  |  | .178 | 126.060 | .430 | .859 | .0202 | .1135 | -.2045 | .2448 |
| GEF | Equal variances assumed | 1.074 | .301 | -.386 | 352 | .350 | .700 | -.0559 | .1448 | -.3406 | .2288 |
|  | Equal variances not assumed |  |  | -.392 | 129.328 | .348 | .695 | -.0559 | .1424 | -.3377 | .2259 |

**T-Test**

| **Group Statistics** | | | | | |
| --- | --- | --- | --- | --- | --- |
|  | Relationship status | N | Mean | Std. Deviation | Std. Error Mean |
| TPMH | Single | 255 | 4.394 | .6702 | .0420 |
|  | In Relationship/Married | 99 | 4.461 | .5571 | .0560 |
| GC | Single | 255 | 4.042 | 1.0537 | .0660 |
|  | In Relationship /Married | 99 | 4.034 | .8483 | .0853 |
| ES | Single | 255 | 4.342 | 1.2570 | .0787 |
|  | In Relationship /Married | 99 | 4.737 | .9603 | .0965 |
| SP | Single | 255 | 4.925 | .9610 | .0602 |
|  | In Relationship /Married | 99 | 4.825 | 1.0700 | .1075 |
| IS | Single | 255 | 4.265 | .8866 | .0555 |
|  | In Relationship /Married | 99 | 4.215 | .7415 | .0745 |
| PGA | Single | 255 | 4.588 | .9138 | .0572 |
|  | In Relationship /Married | 99 | 4.674 | .8156 | .0820 |
| GEF | Single | 255 | 4.232 | 1.1424 | .0715 |
|  | In Relationship /Married | 99 | 4.401 | 1.1033 | .1109 |

| **Independent Samples Test** | | | | | | | | | | | | |
| --- | --- | --- | --- | --- | --- | --- | --- | --- | --- | --- | --- | --- |
|  | | Levene's Test for Equality of Variances | | t-test for Equality of Means | | | | | | | | |
|  |  | F | Sig. | t | df | Significance | | Mean Difference | Std. Error Difference | 95% Confidence Interval of the Difference | |  |
|  |  |  |  |  |  | One-Sided p | Two-Sided p |  |  | Lower | Upper |  |
| TPMH | Equal variances assumed | 4.091 | .044 | -.881 | 352 | .189 | .379 | -.0669 | .0759 | -.2161 | .0823 |  |
|  | Equal variances not assumed |  |  | -.956 | 213.105 | .170 | .340 | -.0669 | .0700 | -.2048 | .0711 |  |
| GC | Equal variances assumed | 5.208 | .023 | .061 | 352 | .476 | .951 | .0072 | .1185 | -.2258 | .2403 |  |
|  | Equal variances not assumed |  |  | .067 | 220.091 | .473 | .947 | .0072 | .1078 | -.2052 | .2197 |  |
| ES | Equal variances assumed | 16.388 | <.001 | -2.825 | 352 | .002 | .005 | -.3954 | .1400 | -.6707 | -.1202 |  |
|  | Equal variances not assumed |  |  | -3.175 | 232.108 | <.001 | .002 | -.3954 | .1245 | -.6408 | -.1500 |  |
| SP | Equal variances assumed | 1.676 | .196 | .846 | 352 | .199 | .398 | .0995 | .1175 | -.1317 | .3306 |  |
|  | Equal variances not assumed |  |  | .807 | 162.821 | .210 | .421 | .0995 | .1232 | -.1439 | .3428 |  |
| IS | Equal variances assumed | 1.249 | .264 | .501 | 352 | .308 | .617 | .0503 | .1005 | -.1473 | .2480 |  |
|  | Equal variances not assumed |  |  | .542 | 211.804 | .294 | .589 | .0503 | .0929 | -.1329 | .2335 |  |
| PGA | Equal variances assumed | 1.801 | .180 | -.817 | 352 | .207 | .414 | -.0859 | .1051 | -.2926 | .1208 |  |
|  | Equal variances not assumed |  |  | -.859 | 198.593 | .196 | .391 | -.0859 | .1000 | -.2830 | .1112 |  |
| GEF | Equal variances assumed | .102 | .749 | -1.260 | 352 | .104 | .209 | -.1689 | .1340 | -.4324 | .0947 |  |
|  | Equal variances not assumed |  |  | -1.280 | 184.255 | .101 | .202 | -.1689 | .1320 | -.4292 | .0915 |  |

**T-Test**

| **Group Statistics** | | | | | |
| --- | --- | --- | --- | --- | --- |
|  | History of Psychiatric Illness | N | Mean | Std. Deviation | Std. Error Mean |
| TPMH | Yes | 13 | 4.077 | .7026 | .1949 |
|  | No | 341 | 4.425 | .6357 | .0344 |
| GC | Yes | 13 | 3.769 | .8577 | .2379 |
|  | No | 341 | 4.050 | 1.0040 | .0544 |
| ES | Yes | 13 | 3.885 | 1.2674 | .3515 |
|  | No | 341 | 4.474 | 1.1872 | .0643 |
| SP | Yes | 13 | 4.969 | 1.1056 | .3066 |
|  | No | 341 | 4.894 | .9892 | .0536 |
| IS | Yes | 13 | 4.262 | .7654 | .2123 |
|  | No | 341 | 4.251 | .8518 | .0461 |
| PGA | Yes | 13 | 3.869 | 1.1463 | .3179 |
|  | No | 341 | 4.640 | .8653 | .0469 |
| GEF | Yes | 13 | 3.800 | 1.1547 | .3203 |
|  | No | 341 | 4.298 | 1.1295 | .0612 |

| **Independent Samples Test** | | | | | | | | | | | | |
| --- | --- | --- | --- | --- | --- | --- | --- | --- | --- | --- | --- | --- |
|  | | Levene's Test for Equality of Variances | | t-test for Equality of Means | | | | | | | | |
|  |  | F | Sig. | t | df | Significance | | Mean Difference | Std. Error Difference | 95% Confidence Interval of the Difference | |  |
|  |  |  |  |  |  | One-Sided p | Two-Sided p |  |  | Lower | Upper |  |
| TPMH | Equal variances assumed | .031 | .860 | -1.932 | 352 | .027 | .054 | -.3483 | .1803 | -.7029 | .0063 |  |
|  | Equal variances not assumed |  |  | -1.760 | 12.760 | .051 | .102 | -.3483 | .1979 | -.7766 | .0800 |  |
| GC | Equal variances assumed | .354 | .552 | -.994 | 352 | .161 | .321 | -.2806 | .2824 | -.8360 | .2748 |  |
|  | Equal variances not assumed |  |  | -1.150 | 13.285 | .135 | .270 | -.2806 | .2440 | -.8066 | .2454 |  |
| ES | Equal variances assumed | .024 | .878 | -1.753 | 352 | .040 | .080 | -.5896 | .3363 | -1.2509 | .0718 |  |
|  | Equal variances not assumed |  |  | -1.650 | 12.816 | .062 | .123 | -.5896 | .3574 | -1.3627 | .1836 |  |
| SP | Equal variances assumed | .085 | .771 | .267 | 352 | .395 | .789 | .0751 | .2807 | -.4770 | .6272 |  |
|  | Equal variances not assumed |  |  | .241 | 12.743 | .407 | .813 | .0751 | .3113 | -.5988 | .7489 |  |
| IS | Equal variances assumed | .120 | .729 | .044 | 352 | .483 | .965 | .0105 | .2399 | -.4614 | .4824 |  |
|  | Equal variances not assumed |  |  | .048 | 13.159 | .481 | .962 | .0105 | .2172 | -.4582 | .4793 |  |
| PGA | Equal variances assumed | 1.599 | .207 | -3.113 | 352 | .001 | .002 | -.7709 | .2477 | -1.2580 | -.2839 |  |
|  | Equal variances not assumed |  |  | -2.399 | 12.527 | .016 | .033 | -.7709 | .3214 | -1.4679 | -.0740 |  |
| GEF | Equal variances assumed | .000 | .997 | -1.558 | 352 | .060 | .120 | -.4977 | .3194 | -1.1259 | .1306 |  |
|  | Equal variances not assumed |  |  | -1.526 | 12.891 | .076 | .151 | -.4977 | .3260 | -1.2026 | .2073 |  |

**T-Test**

| **Group Statistics** | | | | | |
| --- | --- | --- | --- | --- | --- |
|  | Received treatment for psychiatric illness | N | Mean | Std. Deviation | Std. Error Mean |
| TPMH | Yes | 14 | 4.157 | .6572 | .1756 |
|  | No | 340 | 4.423 | .6386 | .0346 |
| GC | Yes | 14 | 3.707 | .8371 | .2237 |
|  | No | 340 | 4.053 | 1.0041 | .0545 |
| ES | Yes | 14 | 4.100 | 1.3393 | .3580 |
|  | No | 340 | 4.467 | 1.1871 | .0644 |
| SP | Yes | 14 | 4.707 | 1.1887 | .3177 |
|  | No | 340 | 4.905 | .9845 | .0534 |
| IS | Yes | 14 | 4.229 | .7141 | .1908 |
|  | No | 340 | 4.252 | .8538 | .0463 |
| PGA | Yes | 14 | 4.179 | 1.1144 | .2978 |
|  | No | 340 | 4.630 | .8740 | .0474 |
| GEF | Yes | 14 | 4.121 | .9609 | .2568 |
|  | No | 340 | 4.286 | 1.1399 | .0618 |

| **Independent Samples Test** | | | | | | | | | | | | |
| --- | --- | --- | --- | --- | --- | --- | --- | --- | --- | --- | --- | --- |
|  | | Levene's Test for Equality of Variances | | t-test for Equality of Means | | | | | | | | |
|  |  | F | Sig. | t | df | Significance | | Mean Difference | Std. Error Difference | 95% Confidence Interval of the Difference | |  |
|  |  |  |  |  |  | One-Sided p | Two-Sided p |  |  | Lower | Upper |  |
| TPMH | Equal variances assumed | .081 | .776 | -1.525 | 352 | .064 | .128 | -.2658 | .1743 | -.6087 | .0771 |  |
|  | Equal variances not assumed |  |  | -1.485 | 14.030 | .080 | .160 | -.2658 | .1790 | -.6497 | .1181 |  |
| GC | Equal variances assumed | .467 | .495 | -1.271 | 352 | .102 | .205 | -.3461 | .2723 | -.8816 | .1894 |  |
|  | Equal variances not assumed |  |  | -1.503 | 14.584 | .077 | .154 | -.3461 | .2303 | -.8381 | .1459 |  |
| ES | Equal variances assumed | .131 | .717 | -1.128 | 352 | .130 | .260 | -.3671 | .3253 | -1.0069 | .2728 |  |
|  | Equal variances not assumed |  |  | -1.009 | 13.854 | .165 | .330 | -.3671 | .3637 | -1.1479 | .4138 |  |
| SP | Equal variances assumed | 1.772 | .184 | -.730 | 352 | .233 | .466 | -.1976 | .2707 | -.7300 | .3349 |  |
|  | Equal variances not assumed |  |  | -.613 | 13.744 | .275 | .550 | -.1976 | .3222 | -.8897 | .4946 |  |
| IS | Equal variances assumed | .034 | .855 | -.103 | 352 | .459 | .918 | -.0238 | .2315 | -.4791 | .4316 |  |
|  | Equal variances not assumed |  |  | -.121 | 14.574 | .453 | .905 | -.0238 | .1964 | -.4434 | .3959 |  |
| PGA | Equal variances assumed | 2.003 | .158 | -1.871 | 352 | .031 | .062 | -.4511 | .2411 | -.9253 | .0230 |  |
|  | Equal variances not assumed |  |  | -1.496 | 13.666 | .079 | .157 | -.4511 | .3016 | -1.0994 | .1972 |  |
| GEF | Equal variances assumed | .539 | .463 | -.532 | 352 | .298 | .595 | -.1645 | .3092 | -.7725 | .4436 |  |
|  | Equal variances not assumed |  |  | -.623 | 14.548 | .272 | .543 | -.1645 | .2642 | -.7290 | .4001 |  |

**One way ANOVA: Age**

| **Descriptives** | | | | | | | | | |
| --- | --- | --- | --- | --- | --- | --- | --- | --- | --- |
|  | | N | Mean | Std. Deviation | Std. Error | 95% Confidence Interval for Mean | | Minimum | Maximum |
|  |  |  |  |  |  | Lower Bound | Upper Bound |  |  |
| TPMH | 18-19 Years | 96 | 4.283 | .6503 | .0664 | 4.152 | 4.415 | 2.2 | 5.4 |
|  | 20-21 Years | 131 | 4.444 | .6556 | .0573 | 4.331 | 4.558 | 2.4 | 5.9 |
|  | 22-23 Years | 84 | 4.412 | .5394 | .0589 | 4.295 | 4.529 | 2.6 | 5.6 |
|  | >23 Years | 43 | 4.605 | .7121 | .1086 | 4.385 | 4.824 | 2.8 | 5.6 |
|  | Total | 354 | 4.412 | .6405 | .0340 | 4.345 | 4.479 | 2.2 | 5.9 |
| GC | 18-19 Years | 96 | 3.902 | 1.0210 | .1042 | 3.695 | 4.109 | 1.4 | 6.0 |
|  | 20-21 Years | 131 | 4.082 | 1.0352 | .0904 | 3.904 | 4.261 | 1.3 | 6.0 |
|  | 22-23 Years | 84 | 3.995 | .9270 | .1011 | 3.794 | 4.196 | 1.3 | 5.7 |
|  | >23 Years | 43 | 4.302 | .9470 | .1444 | 4.011 | 4.594 | 1.3 | 6.0 |
|  | Total | 354 | 4.040 | .9993 | .0531 | 3.935 | 4.144 | 1.3 | 6.0 |
| ES | 18-19 Years | 96 | 4.316 | 1.2463 | .1272 | 4.063 | 4.568 | 1.3 | 6.0 |
|  | 20-21 Years | 131 | 4.380 | 1.2658 | .1106 | 4.161 | 4.599 | 1.0 | 6.0 |
|  | 22-23 Years | 84 | 4.575 | 1.0166 | .1109 | 4.354 | 4.796 | 1.4 | 6.0 |
|  | >23 Years | 43 | 4.740 | 1.1329 | .1728 | 4.391 | 5.088 | 2.1 | 6.0 |
|  | Total | 354 | 4.453 | 1.1935 | .0634 | 4.328 | 4.577 | 1.0 | 6.0 |
| SP | 18-19 Years | 96 | 4.866 | .9601 | .0980 | 4.671 | 5.060 | 1.4 | 6.0 |
|  | 20-21 Years | 131 | 4.917 | 1.0355 | .0905 | 4.738 | 5.096 | 1.0 | 6.0 |
|  | 22-23 Years | 84 | 4.883 | .9525 | .1039 | 4.677 | 5.090 | 1.7 | 6.0 |
|  | >23 Years | 43 | 4.933 | 1.0350 | .1578 | 4.614 | 5.251 | 2.1 | 6.0 |
|  | Total | 354 | 4.897 | .9921 | .0527 | 4.793 | 5.001 | 1.0 | 6.0 |
| IS | 18-19 Years | 96 | 4.268 | .8050 | .0822 | 4.105 | 4.431 | 1.8 | 5.9 |
|  | 20-21 Years | 131 | 4.240 | .9828 | .0859 | 4.071 | 4.410 | 1.2 | 11.0 |
|  | 22-23 Years | 84 | 4.237 | .7050 | .0769 | 4.084 | 4.390 | 2.6 | 6.3 |
|  | >23 Years | 43 | 4.277 | .7755 | .1183 | 4.038 | 4.515 | 2.4 | 5.6 |
|  | Total | 354 | 4.251 | .8478 | .0451 | 4.163 | 4.340 | 1.2 | 11.0 |
| PGA | 18-19 Years | 96 | 4.409 | .9078 | .0926 | 4.225 | 4.593 | 2.1 | 6.0 |
|  | 20-21 Years | 131 | 4.682 | .8698 | .0760 | 4.531 | 4.832 | 1.7 | 6.0 |
|  | 22-23 Years | 84 | 4.600 | .8478 | .0925 | 4.416 | 4.784 | 2.4 | 6.0 |
|  | >23 Years | 43 | 4.874 | .8992 | .1371 | 4.598 | 5.151 | 2.8 | 6.0 |
|  | Total | 354 | 4.612 | .8871 | .0472 | 4.519 | 4.705 | 1.7 | 6.0 |
| GEF | 18-19 Years | 96 | 3.906 | 1.2825 | .1309 | 3.646 | 4.166 | 1.0 | 6.0 |
|  | 20-21 Years | 131 | 4.430 | 1.0171 | .0889 | 4.254 | 4.606 | 1.0 | 6.0 |
|  | 22-23 Years | 84 | 4.289 | 1.0537 | .1150 | 4.061 | 4.518 | 1.0 | 6.0 |
|  | >23 Years | 43 | 4.635 | 1.0708 | .1633 | 4.305 | 4.964 | 2.3 | 6.0 |
|  | Total | 354 | 4.279 | 1.1326 | .0602 | 4.161 | 4.398 | 1.0 | 6.0 |

| **ANOVA** | | | | | | |
| --- | --- | --- | --- | --- | --- | --- |
|  | | Sum of Squares | df | Mean Square | F | Sig. |
| TPMH | Between Groups | 3.322 | 3 | 1.107 | 2.739 | .043 |
|  | Within Groups | 141.504 | 350 | .404 |  |  |
|  | Total | 144.825 | 353 |  |  |  |
| GC | Between Groups | 5.189 | 3 | 1.730 | 1.743 | .158 |
|  | Within Groups | 347.337 | 350 | .992 |  |  |
|  | Total | 352.526 | 353 |  |  |  |
| ES | Between Groups | 7.287 | 3 | 2.429 | 1.716 | .163 |
|  | Within Groups | 495.535 | 350 | 1.416 |  |  |
|  | Total | 502.823 | 353 |  |  |  |
| SP | Between Groups | .216 | 3 | .072 | .073 | .975 |
|  | Within Groups | 347.251 | 350 | .992 |  |  |
|  | Total | 347.467 | 353 |  |  |  |
| IS | Between Groups | .086 | 3 | .029 | .040 | .989 |
|  | Within Groups | 253.658 | 350 | .725 |  |  |
|  | Total | 253.744 | 353 |  |  |  |
| PGA | Between Groups | 7.551 | 3 | 2.517 | 3.260 | .022 |
|  | Within Groups | 270.259 | 350 | .772 |  |  |
|  | Total | 277.810 | 353 |  |  |  |
| GEF | Between Groups | 21.771 | 3 | 7.257 | 5.892 | <.001 |
|  | Within Groups | 431.068 | 350 | 1.232 |  |  |
|  | Total | 452.839 | 353 |  |  |  |

**One way ANOVA: field of study**

| **Descriptives** | | | | | | | | | |
| --- | --- | --- | --- | --- | --- | --- | --- | --- | --- |
|  | | N | Mean | Std. Deviation | Std. Error | 95% Confidence Interval for Mean | | Minimum | Maximum |
|  |  |  |  |  |  | Lower Bound | Upper Bound |  |  |
| TPMH | Pharmacy | 61 | 4.284 | .6380 | .0817 | 4.120 | 4.447 | 3.0 | 5.6 |
|  | Optometry | 40 | 4.238 | .7149 | .1130 | 4.009 | 4.466 | 2.6 | 5.6 |
|  | Nursing | 60 | 4.425 | .6150 | .0794 | 4.266 | 4.584 | 2.8 | 5.6 |
|  | HND | 49 | 4.588 | .6333 | .0905 | 4.406 | 4.770 | 2.2 | 5.9 |
|  | Medicine | 92 | 4.533 | .5746 | .0599 | 4.414 | 4.652 | 2.8 | 5.6 |
|  | Medical Sciences | 52 | 4.306 | .6743 | .0935 | 4.118 | 4.493 | 2.4 | 5.5 |
|  | Total | 354 | 4.412 | .6405 | .0340 | 4.345 | 4.479 | 2.2 | 5.9 |
| GC | Pharmacy | 61 | 3.974 | 1.0276 | .1316 | 3.711 | 4.237 | 1.3 | 6.0 |
|  | Optometry | 40 | 3.938 | 1.1233 | .1776 | 3.578 | 4.297 | 1.3 | 5.9 |
|  | Nursing | 60 | 4.168 | 1.0431 | .1347 | 3.899 | 4.438 | 2.0 | 5.8 |
|  | HND | 49 | 4.010 | 1.0203 | .1458 | 3.717 | 4.303 | 1.3 | 6.0 |
|  | Medicine | 92 | 4.077 | .9445 | .0985 | 3.882 | 4.273 | 1.3 | 6.0 |
|  | Medical Sciences | 52 | 4.008 | .9152 | .1269 | 3.753 | 4.262 | 1.9 | 5.7 |
|  | Total | 354 | 4.040 | .9993 | .0531 | 3.935 | 4.144 | 1.3 | 6.0 |
| ES | Pharmacy | 61 | 4.187 | 1.2398 | .1587 | 3.869 | 4.504 | 1.4 | 6.0 |
|  | Optometry | 40 | 4.405 | 1.1323 | .1790 | 4.043 | 4.767 | 2.1 | 6.0 |
|  | Nursing | 60 | 4.595 | 1.2335 | .1592 | 4.276 | 4.914 | 1.0 | 6.0 |
|  | HND | 49 | 4.455 | 1.1560 | .1651 | 4.123 | 4.787 | 1.3 | 6.0 |
|  | Medicine | 92 | 4.676 | 1.1590 | .1208 | 4.436 | 4.916 | 1.4 | 6.0 |
|  | Medical Sciences | 52 | 4.238 | 1.1869 | .1646 | 3.908 | 4.569 | 1.6 | 6.0 |
|  | Total | 354 | 4.453 | 1.1935 | .0634 | 4.328 | 4.577 | 1.0 | 6.0 |
| SP | Pharmacy | 61 | 4.918 | .7647 | .0979 | 4.722 | 5.114 | 2.6 | 6.0 |
|  | Optometry | 40 | 4.765 | .9736 | .1539 | 4.454 | 5.076 | 2.3 | 6.0 |
|  | Nursing | 60 | 4.615 | 1.1653 | .1504 | 4.314 | 4.916 | 1.0 | 6.0 |
|  | HND | 49 | 5.224 | .8087 | .1155 | 4.992 | 5.457 | 2.9 | 6.0 |
|  | Medicine | 92 | 4.965 | .9482 | .0989 | 4.769 | 5.162 | 2.1 | 6.0 |
|  | Medical Sciences | 52 | 4.869 | 1.1788 | .1635 | 4.541 | 5.197 | 1.1 | 6.0 |
|  | Total | 354 | 4.897 | .9921 | .0527 | 4.793 | 5.001 | 1.0 | 6.0 |
| IS | Pharmacy | 61 | 4.172 | .7634 | .0977 | 3.977 | 4.368 | 2.3 | 5.8 |
|  | Optometry | 40 | 4.025 | .8211 | .1298 | 3.762 | 4.288 | 1.8 | 6.0 |
|  | Nursing | 60 | 4.202 | .8031 | .1037 | 3.994 | 4.409 | 1.2 | 5.9 |
|  | HND | 49 | 4.571 | 1.1724 | .1675 | 4.235 | 4.908 | 2.6 | 11.0 |
|  | Medicine | 92 | 4.338 | .6858 | .0715 | 4.196 | 4.480 | 2.6 | 5.8 |
|  | Medical Sciences | 52 | 4.121 | .8458 | .1173 | 3.886 | 4.357 | 1.9 | 6.3 |
|  | Total | 354 | 4.251 | .8478 | .0451 | 4.163 | 4.340 | 1.2 | 11.0 |
| PGA | Pharmacy | 61 | 4.398 | .8523 | .1091 | 4.180 | 4.617 | 2.3 | 6.0 |
|  | Optometry | 40 | 4.255 | 1.0818 | .1710 | 3.909 | 4.601 | 2.2 | 6.0 |
|  | Nursing | 60 | 4.723 | .7860 | .1015 | 4.520 | 4.926 | 2.9 | 6.0 |
|  | HND | 49 | 4.810 | .8709 | .1244 | 4.560 | 5.060 | 2.4 | 6.0 |
|  | Medicine | 92 | 4.798 | .8331 | .0869 | 4.625 | 4.970 | 2.1 | 6.0 |
|  | Medical Sciences | 52 | 4.492 | .8643 | .1199 | 4.252 | 4.733 | 1.7 | 6.0 |
|  | Total | 354 | 4.612 | .8871 | .0472 | 4.519 | 4.705 | 1.7 | 6.0 |
| GEF | Pharmacy | 61 | 4.059 | 1.0228 | .1310 | 3.797 | 4.321 | 1.3 | 6.0 |
|  | Optometry | 40 | 4.213 | 1.1407 | .1804 | 3.848 | 4.577 | 1.8 | 6.0 |
|  | Nursing | 60 | 4.237 | 1.1989 | .1548 | 3.927 | 4.546 | 1.3 | 6.0 |
|  | HND | 49 | 4.508 | 1.2342 | .1763 | 4.154 | 4.863 | 1.0 | 6.0 |
|  | Medicine | 92 | 4.429 | 1.1401 | .1189 | 4.193 | 4.665 | 1.0 | 6.0 |
|  | Medical Sciences | 52 | 4.158 | 1.0334 | .1433 | 3.870 | 4.445 | 1.8 | 6.0 |
|  | Total | 354 | 4.279 | 1.1326 | .0602 | 4.161 | 4.398 | 1.0 | 6.0 |

| **ANOVA** | | | | | | |
| --- | --- | --- | --- | --- | --- | --- |
|  | | Sum of Squares | df | Mean Square | F | Sig. |
| TPMH | Between Groups | 5.672 | 5 | 1.134 | 2.837 | .016 |
|  | Within Groups | 139.153 | 348 | .400 |  |  |
|  | Total | 144.825 | 353 |  |  |  |
| GC | Between Groups | 1.901 | 5 | .380 | .377 | .864 |
|  | Within Groups | 350.626 | 348 | 1.008 |  |  |
|  | Total | 352.526 | 353 |  |  |  |
| ES | Between Groups | 12.594 | 5 | 2.519 | 1.788 | .115 |
|  | Within Groups | 490.229 | 348 | 1.409 |  |  |
|  | Total | 502.823 | 353 |  |  |  |
| SP | Between Groups | 11.219 | 5 | 2.244 | 2.322 | .043 |
|  | Within Groups | 336.248 | 348 | .966 |  |  |
|  | Total | 347.467 | 353 |  |  |  |
| IS | Between Groups | 9.173 | 5 | 1.835 | 2.611 | .025 |
|  | Within Groups | 244.571 | 348 | .703 |  |  |
|  | Total | 253.744 | 353 |  |  |  |
| PGA | Between Groups | 14.473 | 5 | 2.895 | 3.825 | .002 |
|  | Within Groups | 263.338 | 348 | .757 |  |  |
|  | Total | 277.810 | 353 |  |  |  |
| GEF | Between Groups | 8.654 | 5 | 1.731 | 1.356 | .240 |
|  | Within Groups | 444.185 | 348 | 1.276 |  |  |
|  | Total | 452.839 | 353 |  |  |  |

**One way ANOVA: Income**

| **Descriptives** | | | | | | | | | |
| --- | --- | --- | --- | --- | --- | --- | --- | --- | --- |
|  | | N | Mean | Std. Deviation | Std. Error | 95% Confidence Interval for Mean | | Minimum | Maximum |
|  |  |  |  |  |  | Lower Bound | Upper Bound |  |  |
| TPMH | R0-R5000 | 103 | 4.348 | .6749 | .0665 | 4.216 | 4.479 | 2.2 | 5.9 |
|  | R5001-R10000 | 79 | 4.480 | .6231 | .0701 | 4.340 | 4.619 | 2.6 | 5.6 |
|  | R10001-R20000 | 76 | 4.462 | .6123 | .0702 | 4.322 | 4.602 | 2.8 | 5.6 |
|  | >R20000 | 96 | 4.388 | .6401 | .0653 | 4.258 | 4.517 | 3.2 | 5.6 |
|  | Total | 354 | 4.412 | .6405 | .0340 | 4.345 | 4.479 | 2.2 | 5.9 |
| GC | R0-R5000 | 103 | 3.922 | 1.0446 | .1029 | 3.718 | 4.126 | 1.3 | 6.0 |
|  | R5001-R10000 | 79 | 4.161 | .8815 | .0992 | 3.963 | 4.358 | 1.3 | 6.0 |
|  | R10001-R20000 | 76 | 4.125 | .9823 | .1127 | 3.901 | 4.349 | 1.3 | 5.7 |
|  | >R20000 | 96 | 3.998 | 1.0511 | .1073 | 3.785 | 4.211 | 1.3 | 6.0 |
|  | Total | 354 | 4.040 | .9993 | .0531 | 3.935 | 4.144 | 1.3 | 6.0 |
| ES | R0-R5000 | 103 | 4.300 | 1.2314 | .1213 | 4.059 | 4.541 | 1.3 | 6.0 |
|  | R5001-R10000 | 79 | 4.713 | 1.1183 | .1258 | 4.462 | 4.963 | 1.4 | 6.0 |
|  | R10001-R20000 | 76 | 4.428 | 1.2575 | .1442 | 4.140 | 4.715 | 1.0 | 6.0 |
|  | >R20000 | 96 | 4.422 | 1.1431 | .1167 | 4.190 | 4.653 | 1.7 | 6.0 |
|  | Total | 354 | 4.453 | 1.1935 | .0634 | 4.328 | 4.577 | 1.0 | 6.0 |
| SP | R0-R5000 | 103 | 4.893 | .9344 | .0921 | 4.711 | 5.076 | 2.1 | 6.0 |
|  | R5001-R10000 | 79 | 4.905 | 1.0195 | .1147 | 4.677 | 5.133 | 1.0 | 6.0 |
|  | R10001-R20000 | 76 | 4.928 | .9975 | .1144 | 4.700 | 5.156 | 1.3 | 6.0 |
|  | >R20000 | 96 | 4.870 | 1.0389 | .1060 | 4.659 | 5.080 | 1.1 | 6.0 |
|  | Total | 354 | 4.897 | .9921 | .0527 | 4.793 | 5.001 | 1.0 | 6.0 |
| IS | R0-R5000 | 103 | 4.296 | 1.0165 | .1002 | 4.097 | 4.495 | 1.2 | 11.0 |
|  | R5001-R10000 | 79 | 4.273 | .6948 | .0782 | 4.118 | 4.429 | 2.4 | 5.9 |
|  | R10001-R20000 | 76 | 4.312 | .8443 | .0968 | 4.119 | 4.505 | 1.8 | 6.3 |
|  | >R20000 | 96 | 4.138 | .7641 | .0780 | 3.983 | 4.292 | 1.9 | 6.0 |
|  | Total | 354 | 4.251 | .8478 | .0451 | 4.163 | 4.340 | 1.2 | 11.0 |
| PGA | R0-R5000 | 103 | 4.560 | .9662 | .0952 | 4.371 | 4.749 | 1.7 | 6.0 |
|  | R5001-R10000 | 79 | 4.657 | .8663 | .0975 | 4.463 | 4.851 | 2.2 | 6.0 |
|  | R10001-R20000 | 76 | 4.674 | .7803 | .0895 | 4.495 | 4.852 | 3.0 | 6.0 |
|  | >R20000 | 96 | 4.581 | .9036 | .0922 | 4.398 | 4.764 | 2.1 | 6.0 |
|  | Total | 354 | 4.612 | .8871 | .0472 | 4.519 | 4.705 | 1.7 | 6.0 |
| GEF | R0-R5000 | 103 | 4.134 | 1.2283 | .1210 | 3.894 | 4.374 | 1.0 | 6.0 |
|  | R5001-R10000 | 79 | 4.228 | 1.2121 | .1364 | 3.956 | 4.499 | 1.3 | 6.0 |
|  | R10001-R20000 | 76 | 4.339 | 1.0506 | .1205 | 4.099 | 4.580 | 1.0 | 6.0 |
|  | >R20000 | 96 | 4.430 | 1.0090 | .1030 | 4.226 | 4.635 | 1.8 | 6.0 |
|  | Total | 354 | 4.279 | 1.1326 | .0602 | 4.161 | 4.398 | 1.0 | 6.0 |

| **ANOVA** | | | | | | |
| --- | --- | --- | --- | --- | --- | --- |
|  | | Sum of Squares | df | Mean Square | F | Sig. |
| TPMH | Between Groups | 1.036 | 3 | .345 | .841 | .472 |
|  | Within Groups | 143.789 | 350 | .411 |  |  |
|  | Total | 144.825 | 353 |  |  |  |
| GC | Between Groups | 3.297 | 3 | 1.099 | 1.102 | .349 |
|  | Within Groups | 349.229 | 350 | .998 |  |  |
|  | Total | 352.526 | 353 |  |  |  |
| ES | Between Groups | 7.879 | 3 | 2.626 | 1.857 | .137 |
|  | Within Groups | 494.943 | 350 | 1.414 |  |  |
|  | Total | 502.823 | 353 |  |  |  |
| SP | Between Groups | .149 | 3 | .050 | .050 | .985 |
|  | Within Groups | 347.318 | 350 | .992 |  |  |
|  | Total | 347.467 | 353 |  |  |  |
| IS | Between Groups | 1.767 | 3 | .589 | .818 | .484 |
|  | Within Groups | 251.977 | 350 | .720 |  |  |
|  | Total | 253.744 | 353 |  |  |  |
| PGA | Between Groups | .816 | 3 | .272 | .344 | .794 |
|  | Within Groups | 276.994 | 350 | .791 |  |  |
|  | Total | 277.810 | 353 |  |  |  |
| GEF | Between Groups | 4.846 | 3 | 1.615 | 1.262 | .287 |
|  | Within Groups | 447.994 | 350 | 1.280 |  |  |
|  | Total | 452.839 | 353 |  |  |  |

**One way ANOVA: Current living status**

| **Descriptives** | | | | | | | | | |
| --- | --- | --- | --- | --- | --- | --- | --- | --- | --- |
|  | | N | Mean | Std. Deviation | Std. Error | 95% Confidence Interval for Mean | | Minimum | Maximum |
|  |  |  |  |  |  | Lower Bound | Upper Bound |  |  |
| TPMH | UNIRES | 264 | 4.404 | .6366 | .0392 | 4.327 | 4.481 | 2.4 | 5.6 |
|  | PRIV/LIVOTHER | 82 | 4.440 | .6601 | .0729 | 4.295 | 4.585 | 2.2 | 5.9 |
|  | LIVPARENT | 8 | 4.400 | .6392 | .2260 | 3.866 | 4.934 | 3.4 | 5.6 |
|  | Total | 354 | 4.412 | .6405 | .0340 | 4.345 | 4.479 | 2.2 | 5.9 |
| GC | UNIRES | 264 | 4.041 | 1.0098 | .0621 | 3.919 | 4.163 | 1.3 | 6.0 |
|  | PRIV/LIVOTHER | 82 | 4.056 | .9987 | .1103 | 3.837 | 4.276 | 1.3 | 6.0 |
|  | LIVPARENT | 8 | 3.825 | .6756 | .2389 | 3.260 | 4.390 | 3.0 | 5.3 |
|  | Total | 354 | 4.040 | .9993 | .0531 | 3.935 | 4.144 | 1.3 | 6.0 |
| ES | UNIRES | 264 | 4.471 | 1.1961 | .0736 | 4.326 | 4.616 | 1.0 | 6.0 |
|  | PRIV/LIVOTHER | 82 | 4.382 | 1.2262 | .1354 | 4.112 | 4.651 | 1.3 | 6.0 |
|  | LIVPARENT | 8 | 4.563 | .7726 | .2732 | 3.917 | 5.208 | 3.4 | 5.7 |
|  | Total | 354 | 4.453 | 1.1935 | .0634 | 4.328 | 4.577 | 1.0 | 6.0 |
| SP | UNIRES | 264 | 4.868 | 1.0039 | .0618 | 4.746 | 4.989 | 1.0 | 6.0 |
|  | PRIV/LIVOTHER | 82 | 4.977 | .9495 | .1049 | 4.768 | 5.185 | 2.3 | 6.0 |
|  | LIVPARENT | 8 | 5.038 | 1.0993 | .3887 | 4.118 | 5.957 | 2.6 | 6.0 |
|  | Total | 354 | 4.897 | .9921 | .0527 | 4.793 | 5.001 | 1.0 | 6.0 |
| IS | UNIRES | 264 | 4.172 | .7862 | .0484 | 4.076 | 4.267 | 1.2 | 5.9 |
|  | PRIV/LIVOTHER | 82 | 4.502 | .9856 | .1088 | 4.286 | 4.719 | 2.4 | 11.0 |
|  | LIVPARENT | 8 | 4.313 | .8951 | .3165 | 3.564 | 5.061 | 2.7 | 5.6 |
|  | Total | 354 | 4.251 | .8478 | .0451 | 4.163 | 4.340 | 1.2 | 11.0 |
| PGA | UNIRES | 264 | 4.625 | .9046 | .0557 | 4.515 | 4.735 | 1.7 | 6.0 |
|  | PRIV/LIVOTHER | 82 | 4.550 | .8462 | .0935 | 4.364 | 4.736 | 2.2 | 6.0 |
|  | LIVPARENT | 8 | 4.813 | .7453 | .2635 | 4.189 | 5.436 | 3.6 | 5.7 |
|  | Total | 354 | 4.612 | .8871 | .0472 | 4.519 | 4.705 | 1.7 | 6.0 |
| GEF | UNIRES | 264 | 4.333 | 1.1301 | .0696 | 4.196 | 4.470 | 1.0 | 6.0 |
|  | PRIV/LIVOTHER | 82 | 4.160 | 1.1158 | .1232 | 3.915 | 4.405 | 1.0 | 6.0 |
|  | LIVPARENT | 8 | 3.725 | 1.2948 | .4578 | 2.643 | 4.807 | 2.0 | 5.8 |
|  | Total | 354 | 4.279 | 1.1326 | .0602 | 4.161 | 4.398 | 1.0 | 6.0 |

| **ANOVA** | | | | | | |
| --- | --- | --- | --- | --- | --- | --- |
|  | | Sum of Squares | df | Mean Square | F | Sig. |
| TPMH | Between Groups | .083 | 2 | .041 | .100 | .905 |
|  | Within Groups | 144.743 | 351 | .412 |  |  |
|  | Total | 144.825 | 353 |  |  |  |
| GC | Between Groups | .391 | 2 | .196 | .195 | .823 |
|  | Within Groups | 352.135 | 351 | 1.003 |  |  |
|  | Total | 352.526 | 353 |  |  |  |
| ES | Between Groups | .600 | 2 | .300 | .210 | .811 |
|  | Within Groups | 502.223 | 351 | 1.431 |  |  |
|  | Total | 502.823 | 353 |  |  |  |
| SP | Between Groups | .906 | 2 | .453 | .459 | .633 |
|  | Within Groups | 346.561 | 351 | .987 |  |  |
|  | Total | 347.467 | 353 |  |  |  |
| IS | Between Groups | 6.879 | 2 | 3.440 | 4.890 | .008 |
|  | Within Groups | 246.865 | 351 | .703 |  |  |
|  | Total | 253.744 | 353 |  |  |  |
| PGA | BetwCorreen Groups | .681 | 2 | .341 | .432 | .650 |
|  | Within Groups | 277.129 | 351 | .790 |  |  |
|  | Total | 277.810 | 353 |  |  |  |
| GEF | Between Groups | 4.401 | 2 | 2.200 | 1.722 | .180 |
|  | Within Groups | 448.439 | 351 | 1.278 |  |  |
|  | Total | 452.839 | 353 |  |  |  |

**Correlations between socio-demographic and health related variables and PMH domains**

| **Correlations** | |  |  |  |  |  |  |  |
| --- | --- | --- | --- | --- | --- | --- | --- | --- |
|  |  | TPMH | GC | ES | SP | IS | PGA | GA |
| Gender | Pearson Correlation | -.111* | -.202** | 0.061 | .142** | -0.078 | -.156** | -.194** |
|  | Sig. (2-tailed) | 0.037 | <.001 | 0.251 | 0.008 | 0.145 | 0.003 | <.001 |
|  | N | 354 | 354 | 354 | 354 | 354 | 354 | 354 |
| Ethnicity | Pearson Correlation | 0.08 | 0.036 | .110* | -0.015 | 0.071 | 0.082 | 0.007 |
|  | Sig. (2-tailed) | 0.132 | 0.494 | 0.039 | 0.775 | 0.184 | 0.122 | 0.894 |
|  | N | 354 | 354 | 354 | 354 | 354 | 354 | 354 |
| Age | Pearson Correlation | .156** | .121* | .133* | 0.026 | 0.02 | .148** | .177** |
|  | Sig. (2-tailed) | 0.003 | 0.023 | 0.012 | 0.629 | 0.713 | 0.005 | <.001 |
|  | N | 354 | 354 | 354 | 354 | 354 | 354 | 354 |
| LevelEdu | Pearson Correlation | 0.096 | 0.021 | 0.059 | 0.045 | 0.059 | .123* | 0.073 |
|  | Sig. (2-tailed) | 0.073 | 0.694 | 0.27 | 0.396 | 0.269 | 0.021 | 0.173 |
|  | N | 354 | 354 | 354 | 354 | 354 | 354 | 354 |
| Bursary | Pearson Correlation | -0.031 | -0.021 | -0.03 | -0.027 | -0.007 | -0.014 | -0.032 |
|  | Sig. (2-tailed) | 0.566 | 0.688 | 0.574 | 0.615 | 0.89 | 0.793 | 0.543 |
|  | N | 354 | 354 | 354 | 354 | 354 | 354 | 354 |
| Relaffil | Pearson Correlation | -0.048 | -0.007 | -0.012 | -.139** | -0.054 | 0.003 | 0.013 |
|  | Sig. (2-tailed) | 0.364 | 0.898 | 0.823 | 0.009 | 0.315 | 0.958 | 0.812 |
|  | N | 354 | 354 | 354 | 354 | 354 | 354 | 354 |
| HouholdInc | Pearson Correlation | 0.021 | 0.026 | 0.016 | -0.006 | -0.062 | 0.011 | 0.103 |
|  | Sig. (2-tailed) | 0.69 | 0.632 | 0.764 | 0.91 | 0.242 | 0.833 | 0.052 |
|  | N | 354 | 354 | 354 | 354 | 354 | 354 | 354 |
| FamRes | Pearson Correlation | 0 | 0.027 | 0.041 | -0.048 | -0.053 | -0.009 | 0.021 |
|  | Sig. (2-tailed) | 0.997 | 0.612 | 0.439 | 0.372 | 0.317 | 0.859 | 0.7 |
|  | N | 354 | 354 | 354 | 354 | 354 | 354 | 354 |
| Relstatus | Pearson Correlation | 0.047 | -0.003 | .149** | -0.045 | -0.027 | 0.044 | 0.067 |
|  | Sig. (2-tailed) | 0.379 | 0.951 | 0.005 | 0.398 | 0.617 | 0.414 | 0.209 |
|  | N | 354 | 354 | 354 | 354 | 354 | 354 | 354 |
| HisPsyIll | Pearson Correlation | 0.102 | 0.053 | 0.093 | -0.014 | -0.002 | .164** | 0.083 |
|  | Sig. (2-tailed) | 0.054 | 0.321 | 0.08 | 0.789 | 0.965 | 0.002 | 0.12 |
|  | N | 354 | 354 | 354 | 354 | 354 | 354 | 354 |
| MedTrePsyIll | Pearson Correlation | 0.081 | 0.068 | 0.06 | 0.039 | 0.005 | 0.099 | 0.028 |
|  | Sig. (2-tailed) | 0.128 | 0.205 | 0.26 | 0.466 | 0.918 | 0.062 | 0.595 |
|  | N | 354 | 354 | 354 | 354 | 354 | 354 | 354 |
| Living | Pearson Correlation | 0.019 | -0.012 | -0.019 | 0.051 | .145** | -0.012 | -0.094 |
|  | Sig. (2-tailed) | 0.728 | 0.824 | 0.716 | 0.342 | 0.006 | 0.822 | 0.077 |
|  | N | 354 | 354 | 354 | 354 | 354 | 354 | 354 |
| ** Correlation is significant at the 0.01 level (2-tailed).  * Correlation is significant at the 0.05 level (2-tailed). | | | | | | | | |
