# Supplementary material for: Investigation of positive mental health levels among faculty of health sciences students at a rural university in South Africa
Source: PLOS Glob Public Health. 2024 Jul 29;4(7):e0002855. doi: 10.1371/journal.pgph.0002855 (PMC11285916; doi:10.1371/journal.pgph.0002855)
Supplement: Attachment — Submitted filename: Reseponse To Reviewers.docx [file pgph.0002855.s006.docx]

**PLoS GLOBAL PUBLIC HEALTH**

**Manuscript Title:** **Investigation of positive mental health levels among faculty of health sciences students at a rural university in South Africa**

**Rebuttal/List of Corrections**

| **Reviewer 1** | **Reviewer’s Comments** | **Author’s response** |
| --- | --- | --- |
| **Prof Saumil Dholakia** | 1. **What is the current breadth, width and depth of positive psychology in South Africa? As the authors might be aware, literature in positive mental health has a large representation in the field of positive psychology and having a section on current best research on positive psychology in students in South Africa will help** | ***Thank you for your comments and suggestions.***  ***A through literature review was conducted and the references you suggested were also considered.***  A section on positive psychology in South Africa was included in the introduction section (Page 3-4, Lines 59-90) covering the background of positive psychology in South Africa, factors influencing it and relevant references were included.  Further, a section on Positive Mental of Health of university students in South Africa was included in the introduction section (Page 5-6, Lines 112-143). This section provided details about existing literature on mental well-being in the university students of South Africa. |
|  | 2. **Are there known/unknown differences between positive mental health in urban and rural communities? Authors will benefit from adding a section on this in Introduction.** | A section on differences in positive mental health between the urban and rural residents of South Africa was included in the introduction section (Page 4-5, Lines 91-111). All the known differences of positive mental health between the residents of urban and rural areas of South Africa were included in-text citing appropriate references. |
|  | 3. **Please consider revising line 58 in introduction: " A PMH for students is very important as it affects their ability to learn, achieve academically, and behave appropriately.". Suggest to limit to the previous objective evidence of existing correlations between positive mental health and outcomes.** | Revised (Page 7, Lines 149-151). |
|  | 4. **Paragraph with lines 56-64: point well made, but the relationship between positive mental health and mental illness is much more complex than just presence of one=absence/limiting the other. refer to the two continua model here** | This statement was justified by including the two continua model proposed by Keyes (2000). Concepts of flourishing and languishing were included in the introduction section. (Page 3, Lines 52-58). |
|  | 5. **Suggest revising lines 69-72 as there is some research, as pointed above in point 1, in this area in South Africa.** | Revised (Page 7, Lines 144-154). |
|  | 6. **Data collection and data analysis: Specifics about who collected the data and who analysis it would help improve robustness of the study. Please consider adhering to the checklist of reporting of survey studies (CROSS) developed by the EQUATOR framework and adding the details in the text accordingly as well as providing the completed checklist to the readers of this journal.** | Data collection section was revised. Specifics about data collection were provided on Page 10, Lines 216-230.  The data analysis section was also revised. Specifics about data analysis were provided on Page 11, Lines 254-264.  The manuscript was revised as per CROSS checklist and highlighted throughout. Completed checklist was attached. |
|  | 7. **Consider revising line 246-247. The gender differences may be much more complex than attributed to hormonal changes in menstruation, including role differences in specific cultures etc. Also, the reference 30, to the best of my knowledge, does not allude to hormonal changes in menstruation as a major factor.** | This statement was revised, and appropriate reference was included (Page 18, Lines 270-273). |
|  | 8. **Regarding validity of PMH instrument, consider adding details of language of communication/administration of the scale (whether English or local language) and whether that was a limitation or not.** | The PMH instrument is a validated questionnaire available only in English. We believe that there is no limitation with its use in the study as the medium of instruction and communication in the university is English (Page 8, Lines 175-178).  In addition, a section on validity and reliability and bias were included in the manuscript as per CROSS checklist. |
|  | 9. **Limitations section: please consider including limitations of survey design itself that specifically apply to this research, for example, sampling error and non-response error, as well as the influence of question wording and format. Additionally, survey results can be affected by respondent motivation, availability, and willingness.** | Limitations section was thoroughly revised and all the concerns you raised were addressed (Page 20-21, Lines 305-320). |
| **Reviewer 2** | **Reviewer’s Comments** | **Author’s response** |
| **Prof Dheeraj Kattula** | 1. **What is the difference between Medicine and Medical Sciences?** | Bachelor of Science in Medical Sciences [B.Sc. (Med. Sci.)] is a 4-year degree offered by the university to the students who wish to follow a career in medical research or laboratory scientists (pathologists, histologist etc.). Whereas Medicine is offered by the university as 6-year degree for the students who wish to pursue career as medical doctors and specialists |
|  | 2. **Were there no other affiliations at all? I suggest you say 'others' rather than say none.** | South Africa has a predominant Christian population (>95%). Unsurprisingly, in Limpopo, the rural province of South Africa, people have traditional and ancestral beliefs. Although the participants may have Christian affiliation, but they choose to respond as ‘none’. We assume there is a trend of atheism in the student population.  ‘None’ was revised to ‘others’ as per your suggestion (Page 13, Table 1). |
|  | 3. **For all these domains, it looks like you have given the mean and standard deviation of score per item. You should be presenting group mean of the scores obtained in the domain and not per item.** | The main objective of this study was to identify the association between socio-demographic and health related variables and PMH domains. Published literature served as a basis for presenting the mean scores per item (variable characteristics) with standard deviation and a p-value. The results from Table 2 were the backbone of the discussion section. Without the item scores and p-value scores our discussion section is null and void.  However, a correlation test was conducted to identify the association between the variables as a group and PMH domains.  We are attaching existing literature (in pdf format) where variables were presented per item while comparing with PMH scores.   |
|  | 4. **There is a lot of scope for studying the correlation between the domains presented here to further understanding about PMH.** | Bivariate correction test was conducted to identify the corrections between socio-demographic and health related variable and PMH domains.  Results were presented in Page 16-17, Table 3, Lines 228-237. |

**Author’s General Remarks (If any):**

The authors have made major revision to the manuscript based on the reviewer’s comments, CROSS checklist, and comprehensive literature review. The corrections were highlighted in yellow throughout the manuscript.

We have completely removed the details of the study site as per our research and ethics committee’s suggestion.

**Submission Date:** 04/01/2024
