# Supplementary material for: Investigation of positive mental health levels among faculty of health sciences students at a rural university in South Africa
Source: PLOS Glob Public Health. 2024 Jul 29;4(7):e0002855. doi: 10.1371/journal.pgph.0002855 (PMC11285916; doi:10.1371/journal.pgph.0002855)
Supplement: Attachment — Submitted filename: Response to Reviewers.docx [file pgph.0002855.s007.docx]

**PLoS GLOBAL PUBLIC HEALTH**

**Manuscript Title:** **Investigation of positive mental health levels among faculty of health sciences students at a rural university in South Africa**

**Rebuttal/List of Corrections**

| **Reviewer 1** | **Reviewer’s Comments** | **Author’s response** |
| --- | --- | --- |
|  | 1. **Much improved version, congratulate the authors to have dived in deep.**   **The manuscript can be reviewed for flow and grammar but otherwise meets the publication criteria and readers of this journal will benefit from this well-conducted survey on positive mental health in South Africa.** | ***Thank you for your comments, suggestions and appreciation.***  The manuscript was once again reviewed for flow and grammar after the suggested corrections. These were highlighted in the manuscript. |
| **Reviewer 2** | **Reviewer’s Comments** | **Author’s response** |
|  | 1. **Table 3 is wrong. You cannot do correlations between categorical variable like gender, race etc with continuous variables from scale scores. Kindly consult a statistics staff and do the needful.**   **You are to compare variables like total PMH between men and women using t test, degree and PMH using ANOVA/Kruskal Wallis test depending on distribution.**  **Please do the needful and resubmit.** | ***Thank you for your comments and suggestions relating to the data analysis.***  We have analysed the data again. Firstly, to identify whether our sample fits a normal distribution, we conducted normality tests with Kolmogorov–Smirnov test and Shapiro–Wilk test. Additionally, Chi-Square Goodness-of-Fit Test was conducted to identify if the frequencies of distribution of categorical variables were different. All the above tests indicated that our data significantly (*p*<0.001) deviated from a normal distribution (Table 1).  Therefore, as per your suggestion, nonparametric tests were conducted to identify the association between PMH domains and socio-demographic and health related variables. Mann–Whitney U test (Independent sample t-test) and Kruskal-Wallis H test (One-way ANOVA) were employed to identify the association between grouping variables and PMH domains (Table 3). |
| **Reviewer 3** | **Reviewer’s Comments** | **Author’s response** |
|  | 1. **Authors have confused the the terms "affect" and "effect" in many places.** | Thank you for your comments and suggestions.  This typo error appeared to have happened with one of the PMH domain ‘Global effect’, which appeared thrice in our manuscript as ‘Global affect’. This was duly corrected and highlighted in yellow in line 207, 237 and 238. |
|  | **Journal requirements** | **Author’s response** |
|  | 1. **Please review your reference list to ensure that it is complete and correct. If you have cited papers that have been retracted, please include the rationale for doing so in the manuscript text, or remove these references and replace them with relevant current references. Any changes to the reference list should be mentioned in the rebuttal letter that accompanies your revised manuscript. If you need to cite a retracted article, indicate the article’s retracted status in the References list and also include a citation and full reference for the retraction notice** | All the references were reviewed and now they are complete. Appropriate page numbers were added, and DOIs/access links were included for each reference.  No references were excluded, and we did not include any papers that were retracted as references in our manuscript. |

**Author’s General Remarks (If any):**

The authors have made minor revision to the manuscript based on the reviewer’s comments. The authors reviewed the manuscript again for spelling errors, grammar, and flow. These corrections were highlighted in yellow throughout the manuscript.

We have completely removed the details of the study site and the location of the study site as per our research and ethics committee’s suggestion.

**Submission Date:** 28/04/2024
