# Supplementary material for: Investigation of positive mental health levels among faculty of health sciences students at a rural university in South Africa
Source: PLOS Glob Public Health. 2024 Jul 29;4(7):e0002855. doi: 10.1371/journal.pgph.0002855 (PMC11285916; doi:10.1371/journal.pgph.0002855)
Supplement: Attachment — Submitted filename: PLoS Global Public Health Rebuttal Template_20240608.docx [file pgph.0002855.s010.docx]

**PLoS GLOBAL PUBLIC HEALTH**

**Manuscript Title:** **Investigation of positive mental health levels among faculty of health sciences students at a rural university in South Africa**

**Rebuttal/List of Corrections**

| **Reviewer** | **Suggested correction** | **Authors’ response/corrections** | **Line/s** |
| --- | --- | --- | --- |
| **Chief Editor suggested corrections** | ‘’Although literature exists on the impact of urbanisation on physical health and mental illness, but very little on psychosocial well-being’’ | *Although literature exists on the impact of urban living on physical health and mental illness, there is very little on psychosocial well-being* | 102-103 |
|  | ‘’A study conducted in the North West Province of South Africa explored the socio-demographic variables and psychosocial well-being of an African group in rural and urban areas using the General Psychological Well-being (GPW) model and the Mental Health Continuum (MHC) model’’ | *A study conducted in the North West Province of South Africa explored the socio-demographic variables and psychosocial well-being of an African group in rural and urban areas using the General Psychological Well-being (GPW) and the Mental Health Continuum (MHC) models* | 103-106 |
|  | ‘’Students reported a combination of mental disorders such as major depression, mania and hypomania, generalised anxiety disorders, panic disorder, alcohol use disorder, and substance use disorder that impacted their academic performance and overall well-being’’ | *Students reported a combination of mental disorders such as major depression, mania, anxiety disorders, panic disorder, alcohol use disorder, and substance use disorder that impacted their academic performance and overall well-being* | 114-116 |
|  | ‘’In response to this alarming trend, it is crucial for higher education institutions to actively address and prioritise the mental health needs of their students by creating a healthier campus environment that supports PMH and well-being’’ | *In response to this alarming trend, it is crucial for higher education institutions to actively address the mental health needs of their students by creating a healthier campus environment that supports PMH and well-being* | 116-119 |
|  | ‘’For the first five domains, participants were requested to mark how much each item describes them on a scale from 1 to 6 (1- ‘Not at all like me’ to 6-’Exactly like me’)’’ | *For the first five domains, participants were requested to mark how much each item describes them on a scale between 1 to 6 (1- ‘Not at all like me’ to 6-’Exactly like me’)* | 207-208 |
|  | More than half of the students were female (59.9%), affiliated to with Christianity (69.2%), and had a household income of less than South African Rand (ZAR) 10000 per month (50.4%, n=182) | *More than half of the students were female (59.9%), affiliated with Christianity (69.2%), and had a household income of less than 10 000 South African Rand (ZAR) per month (50.4%, n=182).* | 279-281 |
|  | ‘’Nonparametric tests were further conducted to identify the association between PMH domains and socio-demographic and health-related variables’’ | *‘’Removed* |  |
|  | ‘’This can be attributed to the location of the university which is in the rural part of South Africa. The university is situated in one of the poorest and rural provinces of South Africa with predominantly black population’’ | *This can be attributed to the location of the university, which is in the rural and poorest part of South Africa with a predominantly black population.* | 231-232 |
|  | ‘’However, most of the students with a low level of PMH were female and this finding is in line with the existing research’’ | *However, most of the students with a low level of PMH were female, which aligns with the findings of previous research* | 236-237 |
|  | ‘’Age was found to be significantly associated with Total PMH, with students between the age of 18-19 years (first year of study) reporting significantly lower levels of PMH compared other age groups in the study in all PMH domains excluding emotional support and interpersonal skills’’ | *Age was found to be significantly associated with total PMH, with students between the ages of 18 and 19 years (first year of study) reporting significantly lower mean scores compared to other age groups in most domains, excluding emotional support and interpersonal skills.* | 249-251 |
|  | ‘’In this study, field of study was found to be significantly associated with total PMH, with students in HND reporting high levels of PMH and in all domains except general coping and emotional support when compared to the students from other fields of study’’ | *In this study, the field of study was found to be significantly associated with total PMH, with students in HND reporting higher mean scores in most domains except general coping and emotional support when compared to the students from other fields of study* | 266-267 |
|  | ‘’Although students who had a history of psychiatric illness and received treatment for such illness reported lower levels of PMH, the findings cannot be justified due to the limited sample’’ | *Despite the fact that students with a history of psychiatric illness and those receiving treatment reported lower levels of PMH, the limited sample makes the findings unjustifiable.* | 288-290 |
|  | ‘’Devi, R.G., Priya, A.J. and Ranjan, M.R., 2021. Effect of Stress on Mental Health. Journal of Pharmaceutical Research International, 33, pp.491-500. doi: <https://doi.org/10.9734/jpri/2021/v33i60A34510>’’ | *Ranjan, M. R., Priya, A. J. and Devi, R. G. (2021) “Effect of Stress on Mental Health”, Journal of Pharmaceutical Research International, 33(60A), pp. 491–500. doi: 10.9734/jpri/2021/v33i60A34510* | 315-316 |
|  | Ooi, P.B., Khor, K.S., Tan, C.C. and Ong, D.L.T., 2022. Depression, anxiety, stress and satisfaction with life: moderating role of interpersonal needs amongst university students. Frontiers in Public Health, 10:958884. Pp.1-16. doi: <https://doi.org/10.3389/fpubh.2022.958884> | Ooi, P. B., Khor, K. S., Tan, C. C. and Ong, D. L. T. 2022. Depression, anxiety, stress, and satisfaction with life: Moderating role of interpersonal needs among university students. *Frontiers in Public Health*, 10, 958884. doi: <https://doi.org/10.3389/fpubh.2022.958884>  The published article is attached. Citation and DOI seems to be correct. |   387-389 |
|  | Seroto, J., 2011. The provision of rural education in three provinces of South Africa since 1994: Implications for school improvement. Journal of Educational Studies, (si-1), pp.138-152. Available from: <https://hdl.handle.net/10520/EJC160134> | Seroto, J. (2011). The provision of rural education in three provinces of South Africa since 1994: Implications for school improvement. Journal of Educational Studies, 2011(si-1), 138-152.  Unfortunately, we could not obtain the DOI for this publication. | 437-439 |
|  | Dessie, Y., Ebrahim, J. and Awoke, T., 2013. Mental distress among university students in Ethiopia: a cross sectional survey. Pan African Medical Journal, 15(1), pp.1-8. doi: <https://doi.org/10.11604/pamj.2013.15.95.2173> | 1. Dessie, Y., Ebrahim, J. and Awoke, T., 2013. Mental distress among university students in Ethiopia: a cross sectional survey. Pan African Medical Journal, 15(1). doi: https://doi.org/10.11604/pamj.2013.15.95.2173  The DOI links to this publication.  Attached for your reference. |   463-465 |
| **Authors’ initiated corrections** | ‘’The impact of apartheid on psychology in South Africa has been profound, with historical and contextual factors shaping the trajectory of psychological work on health issues’’ | *Apartheid has had a profound impact on South African psychology, with historical and contextual factors shaping the trajectory of psychological work on health issues* | 77-78 |
|  | ‘’The research team obtained authorisation from the authors of the PMH instrument to use in this study. This covers a copyright grant and the use of the scoring algorithm’’. | The research team obtained authorisation from the authors of the PMH instrument to use it in this study. This includes a copyright grant and the scoring algorithm. | 186-188 |
|  | ‘’Hence, the results cannot be generalised to the entire student population in South Africa’’ | *Therefore, we cannot generalise the results to the entire student population in South Africa.* | 281-282 |

**Author’s General Remarks (If any):**

We appreciate your recommendations and revisions. We have acknowledged your revisions and highlighted all the modifications made in the manuscript.

Table 3 and its corresponding content have been removed from the previous version.

All the references were checked again, correct citation from Google scholar was included. One reference featured twice in the previous version of the manuscript; it was removed. Therefore, the reference numbers have changed.

We have thoroughly examined the manuscript once more, rephrased few sentences, and rectified the grammatical errors.

**Submission Date:** 08/06/2024
