# Supplementary material for: Investigation of positive mental health levels among faculty of health sciences students at a rural university in South Africa
Source: PLOS Glob Public Health. 2024 Jul 29;4(7):e0002855. doi: 10.1371/journal.pgph.0002855 (PMC11285916; doi:10.1371/journal.pgph.0002855)
Supplement: Attachment — Submitted filename: PGPH-D-23-02265 REVIEWER comments.pdf [file pgph.0002855.s005.pdf]

PLOS Global Public Health

Investigation of positive mental health levels among faculty of health sciences students at a rural university in South Africa

--Manuscript Draft--

**Manuscript Number:**	PGPH-D-23-02265
**Article Type:**	Research Article
**Full Title:**	Investigation of positive mental health levels among faculty of health sciences students at a rural university in South Africa
**Short Title:**	Investigation of positive mental health levels in students at a university in South Africa
**Corresponding Author:**	Rajesh Vagiri, PhD<br>University of Limpopo - Turfloop Campus: University of Limpopo<br>Polokwane, Limpopo SOUTH AFRICA
**Order of Authors:**	Rajesh Vikram Vagiri, PhD
	Phuty Elizabeth Leboho, BPharm
	Lokwene Katlego Desry, BPharm
	Machaka Khutso, BPharm
	Mbedzi Pfunzo, BPharm
**Keywords:**	Mental well-being; Positive Mental Health; interventions; outcomes; stressors
**Abstract:**	One out of every four people during their lives can be affected by mental health problems altering their functioning, behaviour, and thinking patterns. There has been an increase in mental health disorders among students worldwide in recent times. Positive mental health (PMH) has gained relevance in today's fast-paced and demanding world, especially for university students as it affects their ability to learn, achieve academically, and behave appropriately. This study aimed to investigate the levels of PMH and identify the association between PMH domains and socio-demographic and health related variables among Faculty of Health Sciences (FHS) students at University of Limpopo. A quantitative, descriptive, and cross-sectional study was conducted. Data was collected using multidimensional PMH Instrument and socio-demographic and health related questionnaire, from 354 undergraduate students who are registered for various programs offered by FHS. Data were and analysed using IBM SPSS® version 29. Ethical clearance to conduct the study was obtained from Turfloop Research and Ethics Committee (TREC) of University of Limpopo. The majority of the students were black (99.2%, n=351), single (72%, n=255), received a study bursary from the government (78.5%, n=278), hailed from a rural area (77.7%, n=275) and residing on the university campus (74.6%, n=246). The Total PMH scores of the participants ranged from 4.24 to 4.97 suggesting moderate to higher PMH levels. Significant differences in mean scores were observed in the Total PMH and domains of PMH across various socio-demographic and health related variables. Gender (p=0.037), age (p=0.043) and field of study (p=0.016) showed significant association with Total PMH score. The results of the study highlighted the multi-dimensionality of mental health and justified the importance of evaluating the domains of PMH in FHS students. The disparities observed across different PMH domains underscore the necessity for embracing innovative approaches to achieve the most effective outcomes to improve the mental health and accurate management of symptoms in students. Understanding and promoting PMH in university students is vital to ensure their holistic development and academic success.
**Additional Information:**	
**Question**	**Response**
**Financial Disclosure**	I declare that I have not received any grants or funding support for the research presented in the manuscript from any organization that may have a vested interest in the outcomes of the study. I am currently employed as a researcher and lecturer at the University of Limpopo, South Africa. My academic position does not involve any

Powered by Editorial Manager® and ProduXion Manager® from Aries Systems Corporation

Enter a financial disclosure statement that describes the sources of funding for the work included in this submission and the role the funder(s) played. This includes grants and any commercial funding of the work or authors.<br><br>This statement will be typeset if the manuscript is accepted for publication.<br><br>*Please review the submission guidelines and the instructions link below for detailed requirements and guidance.*	financial associations that could be perceived as a conflict of interest with the content of the manuscript.
**Competing Interests**<br><br>On behalf of all authors, disclose any competing interests that could be perceived to bias this work.<br><br>This statement will be typeset if the manuscript is accepted for publication.<br><br>*Please review the instructions link below and PLOS Global Public Health's competing interests policy to determine what information must be disclosed at submission.*	I declare that I have not received any grants or financial support for the research outlined in the manuscript from any organization that could be perceived as having a conflicting interest in the outcomes of the study.<br>I am currently employed as a researcher and lecturer at the University of Limpopo, South Africa. My academic position is not linked to any entity that might present a competing interest concerning the content of the manuscript.
**Data Availability**<br><br>Before publication, Authors are required to make fully available and without restriction all data underlying their findings. Please see our PLOS Data Policy page for detailed information on this policy.<br><br>A **Data Availability Statement**, detailing where the data can be accessed, is required at first submission. Insert your Data Availability Statement in the box below.	I would like to emphasize our commitment to transparency and reproducibility in research. I'm willing to make all the data available if requested. we aim to contribute to the scientific community's ability to verify and build upon the findings presented in the manuscript.

Powered by Editorial Manager® and ProduXion Manager® from Aries Systems Corporation

Please see the data reporting section of our submission guidelines for instructions on what you need to include in your Data Availability Statement.<br><br>This statement will be typeset if the manuscript is accepted for publication.<br><br>*PLOS allows rare exemptions to address legal and ethical concerns. If you have legal or ethical restrictions, please use the box below to detail these in full sentences for the Journal team to consider.*	

Manuscript

Click here to access/download;Manuscript;Vagiri et al_Investigation of positive mental health levels.docx

Investigation of positive mental health levels among faculty of health sciences students at a rural university in South Africa

Rajesh Vikram Vagiri[1¶]* Phuty Elizabeth Leboho [1¶], Lokwene Katlego Desry[1¶], Machaka Khutso[1¶] & Mbedzi Pfunzo[1¶]

[1]Department of Pharmacy, Faculty of Health Sciences, University of Limpopo, Mankweng, Limpopo Province, South Africa 0727

[*]Corresponding author: E-mail: rajesh.vagiri@ul.ac.za

Abstract

One out of every four people during their lives can be affected by mental health problems altering their functioning, behaviour, and thinking patterns. There has been an increase in mental health disorders among students worldwide in recent times. Positive mental health (PMH) has gained relevance in today's fast-paced and demanding world, especially for university students as it affects their ability to learn, achieve academically, and behave appropriately. This study aimed to investigate the levels of PMH and identify the association between PMH domains and socio-demographic and health related variables among Faculty of Health Sciences (FHS) students at University of Limpopo. A quantitative, descriptive, and cross-sectional study was conducted. Data was collected using multidimensional PMH Instrument and socio-demographic and health related questionnaire, from 354 undergraduate students who are registered for various programs offered by FHS. Data were and analysed using IBM SPSS® version 29. Ethical clearance to conduct the study was obtained from Turfloop Research and Ethics Committee (TREC) of University of Limpopo. The majority of the students were black (99.2%, n=351), single (72%, n=255), received a study bursary from the government (78.5%, n=278), hailed from a rural area (77.7%, n=275) and residing on the university campus (74.6%, n=246). The Total PMH scores of the participants ranged from 4.24 to 4.97 suggesting moderate to higher PMH levels. Significant

differences in mean scores were observed in the Total PMH and domains of PMH across various socio-demographic and health related variables. Gender (p=0.037), age (p=0.043) and field of study (p=0.016) showed significant association with Total PMH score. The results of the study highlighted the multi-dimensionality of mental health and justified the importance of evaluating the domains of PMH in FHS students. The disparities observed across different PMH domains underscore the necessity for embracing innovative approaches to achieve the most effective outcomes to improve the mental health and accurate management of symptoms in students. Understanding and promoting PMH in university students is vital to ensure their holistic development and academic success.

KEYWORDS: Mental well-being, Positive Mental Health, interventions, outcomes, stressors

Introduction

Mental health is, to date, considered a neglected area in developing countries. Mental health problems can affect one out of every four people during their lives, by altering functioning, behaviour, and thinking patterns [1-2]. In today's fast paced society, there is an increasing recognition of the importance of mental health and well-being [3-4]. Mental health refers to a state of emotional, psychological, and social well-being in which individuals can cope with the stresses of life, work productively, and make meaningful contributions to their communities [5]. Well-being has been proposed as a combination of two traditional approaches: “hedonic”- and “eudemonic” concepts. The hedonic concept of well-being has been defined as “high positive effect, low negative effect and high life satisfaction” which focuses on feelings of individuals towards life. Whereas eudemonic concept focuses on functioning in life which include the dimensions of self-acceptance, personal growth, autonomy, relationships, and environmental mastery [6].

In a fast-paced and demanding society, positive mental health has become a topic of increasing importance, particularly among university students [7-8]. The concept of positive mental health goes beyond the absence of mental illness and encompasses the cultivation of emotional resilience, well-being, and overall psychological flourishing [4, 9-10]. A PMH for students is very important as it affects their ability to learn, achieve academically, and behave appropriately. As they navigate the challenges of higher education, students often face various stressors such as academic pressure, financial burdens, and social expectations. These stressors can have a significant impact on their overall well-being and academic performance [11-13].

Globally, there is a rise in mental disorders among students in recent years. According to WHO World Mental Health International College Student project conducted at 19 colleges in eight developed and developing countries revealed that the colleges are contending with increased rates of mental health conditions. Students reported a combination of mental disorders such as major depression, mania/hypomania, generalized anxiety disorder, panic disorder, alcohol use disorder, and substance use disorder that impacted their academic performance and overall well-being [14]. In response to this alarming trend, it is crucial for higher education institutions to actively address and prioritize the mental health needs of their students by creating a healthier campus environment that supports positive mental health and well-being [15-17].

Additionally, there is a growing recognition of the need for preventive mental health practices among university students [7-8]. As students navigate the pressures of academics, social interactions, and personal development, it is crucial to understand and support their positive mental health. [18-19]. Understanding and promoting positive mental health in university students is vital to ensure their holistic development and academic success [20]. Very limited studies were conducted in South Africa to measure PMH among university students and we were the first to investigate the levels of PMH and its association with socio-demographic and health related variables in a rural university of South Africa.

Methods

Study design and population

A cross-sectional study was conducted among Faculty of Health Sciences (FHS) students at University of Limpopo in South Africa. A cross-sectional research design was used because the design allows studies to collect data to make inferences about a population of interest at one point in time. This study followed a descriptive and quantitative approach that included FHS undergraduate students from University of Limpopo, who were registered in the fields of medicine, medical sciences, pharmacy, optometry, nursing sciences, and human nutrition and dietetics (HND).

A sample of 354 students was proportionally distributed between various fields of study: Pharmacy (n=31), Optometry (n=40), Human Nutrition & Dietetics (HND) (n=49), Medicine (n=92), Medical Sciences (n=52) and Nursing Sciences (n=60) to achieve the desired sample.

Data collection tools

Socio-demographic and health related questionnaire

The socio-demographic and health related information such as gender, race, age, field of study, religious affiliation, household income, family residence, relationship status, history of psychiatric illness, whether they have taken any medicine for treating psychiatric illness and who they currently living with were obtained from the participants.

Positive Mental Health Instrument

The researchers used multi-dimensional PMH instrument developed by Vaingankar et al., 2011 [21]. The PMH instrument is a self-administered tool that covers all key and culturally appropriate domains of mental health that can be applied to compare levels of mental health across different populations. This instrument has 57 questions which include six domains:

The domains include:

1. **General coping** refers to individuals' response and coping strategies during stressful situations and their ability to think positively and participate in selected activities.
2. **Emotional support** is key for helping people cope with difficult situations in life and promising feeling loved and wanted. A willingness to share the burden with others and is important to obtain compassionate advice and care.
3. **Spirituality** covers both spiritual and religious practices and beliefs that influence individual faith and behaviour in life. This contributes to PMH as a coping mechanism and means of establishing strong social support and network.
4. **Interpersonal skills** are associated with all aspects of mental health and are essential in helping the individual to develop and maintain good relationships, which in turn provide the support and network needed during times of distress.
5. **Personal Growth and Autonomy** is knowing one's goals and ways to achieve them is a sign of good mental health. It reflects the level of confidence, freedom, sense of purpose, and the ability to self-evaluate and make decisions.
6. **Global Effect** is the experience of a positive mood is a sign of mental health. Calm, happy and enthusiasm means emotional stability and full of energy.

For the first five domains, participants were requested to mark how much each item describes them on a scale from1 to 6 (1- 'Not at all like me' to 6-'Exactly like me'). For the

‘Global affect’ domain, they were requested to indicate ‘how often over the past four weeks they felt - calm, peaceful, relaxed and enthusiastic’ using a 5-point response scale (1- ‘Never or very rarely’ to 5- ‘Very often or always’). Domain specific scores were calculated by summing the scores of the respective items and dividing by the number of items in each domain.

Data collection

Data were collected over a period of three weeks from September 2023 to October 2023. The students who met the inclusion criteria were conveniently enrolled for the study. On the day of data collection, the researchers briefed the students about the study after their theory and practical classes and explained the purpose and importance of this study and requested their participation. They were provided with a study information leaflet to provide additional information about the study. The questionnaires were administered after the participants had given written consent to participate in the study.

Data analysis

Responses from the questionnaires were exported to Microsoft Office Excel® and analysed using IBMs Statistical package for the Social Studies (SPSS) version 29. The PMH scores were calculated using the scoring script provided by the authors of PMH instrument. Recorded item scores for the questions specific to each domain were pooled together and divided by number of questions to obtain the domain scores. The PMH scores ranged from: 1(lower PMH) to 6 (higher PMH). The statistical significance for the study was set at $p \leq 0.05$ using 2-sided tests. Independent t-tests and Analysis of Variance (ANOVA) were used to establish differences in mean scores between Total PMH and specific domains, and socio-demographic and health related variables.

Ethical considerations

Ethical clearance (TREC/518/2023:UG) to conduct the study was obtained from the University of Limpopo Turfloop Research Committee (TREC). Permission to conduct the study was obtained from the Registrar of University of Limpopo. Authorisation to use the PMH instrument was obtained from the National Institute of Mental Health, Singapore.

A participant information leaflet was provided to the students informing them of the objectives of the study, that participation in the study is voluntary, that they have the right to withdraw from the study at any time without providing reasons and that their identity will remain anonymous. All the concerns of the prospective participants regarding the study were clarified before commencement of data collection. All the participants have provided written consent before participation in the study. Adherence to all ethical standards and ethical research practices were maintained in the study. All the records and data were kept in a safe and secured place to maintain confidentiality.

Results

The study population comprised of 354 FHS students from first year to final year with a mean age of 20.6 years. The socio-demographic characteristics of the respondents are shown in Table 1. The majority of the students were black (99.2%, n=351), single (72%, n=255), received study bursary from the government (78.5%, n=278), hailed from a rural area (77.7%, n=275) and residing in the university campus (74.6%, n=246). More than half of the students were female (59.9%), affiliated to Christianity (69.2%), and had a household income less than ZAR 10000 per month (50.5%, n=152). The majority of the students indicated that they did not have a history of any psychiatric illness (96%, n=340) or have taken any psychiatric medicines before (96.3%, n=341).

Table 1: Socio-demographic characteristics of the sample (n=354)

Socio-demographic characteristics		Number of students (n=354)	Percentage (%)
Gender	Male	142	40.1
	Female	212	59.9
Race	Black	351	99.2
	White	3	0.8
Age (in years)	18-19	96	27.2
	20-21	131	37.0
	22-23	84	23.7
	>23	43	12.1
Degree	Pharmacy	61	17.2
	Optometry	40	11.3
	Human Nutrition & Dietetics	49	13.8
	Medicine	92	26.0
	Medical Sciences	52	14.6
	Nursing	60	16.9
Bursary	Yes	278	78.5
	No	76	21.5
Religious affiliation	Christian	245	69.2
	None	109	30.8
Household income (in South African Rands per month)	R 0- R500	100	28.2
	R 5001-R10000	79	22.3
	R 10001-R20000	73	21.4
	>R 20000	96	27.1
Family residence	Rural	275	77.7
	Urban	79	22.3
Current living status	In university residence	246	74.5
	Private residence	82	23.2
	Living with parents	8	2.3
Relationship status	Single	255	72.0
	In a relationship/ Married	99	28.0
History of psychiatric illness	Yes	14	4.0
	No	340	96.0
Have you taken any psychiatric medicines before?	Yes	13	3.7
	No	341	96.3

Total PMH and domain-specific scores by socio-demographic and health related variables

The mean total PMH and domain-specific scores by socio-demographic and health related variables are shown in Table 2. The total PMH scores ranged from 4.24 to 4.97 suggesting that the students of FHS reporting a moderate to higher level of PMH. Significant differences were observed in the total PMH and domain mean scores across various socio-demographic and health related variables. Gender (p=0.037), age (p=0.043) and field of study (p=0.016) had a significant influence on total PMH score. In case of general coping domain, significant differences in mean

scores were observed with gender ($p<0.001$), where males reported significantly higher scores as compared to females. Significant differences in mean scores regarding emotional support domain were observed with race ($p=0.039$) and relationship status ($p=0.02$). There is a significant difference in mean scores with field of study ($p=0.043$), gender ($p=0.008$) and religious affiliation ($p=0.009$) on spirituality. In the case of interpersonal skills domain, significant difference in mean scores was observed with field of study ($p=0.025$) and current living status ($p=0.008$). With respect to personal growth of autonomy significant differences in mean scores were observed in age ($p=0.022$), field of study ($p=0.002$), history of psychiatric illness ($p=0.002$) and gender ($p=0.003$). In global affect, the significant difference in mean score were observed with age ($p<0.001$) and gender ($p<0.001$).

Table 2: Total PMH and domain scores by socio-demographic characteristics

Socio-demographic Characteristics		Total PMH		General coping		Emotional support		Spirituality		Interpersonal skills		Personal growth and autonomy		Global effect	
		Mean (±SD)	P	Mean (±SD)	P	Mean (±SD)	p	Mean (±SD)	p	Mean (±SD)	P	Mean (±SD)	p	Mean (±SD)	p
Gender	Male	4.50 (0.68)	0.037*	4.29 (0.91)	<0.001*	4.36 (1.12)	0.251	4.73 (1.03)	0.008*	4.33 (0.87)	0.145	4.78 (0.96)	0.003*	4.55 (1.06)	<0.001
	Female	4.35 (0.61)		3.89 (1.03)		4.51 (1.25)		5.01 (0.95)		4.20 (0.83)		4.50 (0.82)		4.10 (1.15)	
Race	Black	4.41 (0.13)	0.132	4.04 (1.00)	0.494	4.44 (1.19)	0.039*	4.90 (0.99)	0.775	4.25 (0.85)	0.184	4.61 (0.89)	0.122	4.28 (1.14)	0.894
	White	4.97 (0.40)		4.43 (0.29)		5.87 (0.15)		4.73 (1.48)		4.90 (0.95)		5.40 (0.66)		4.37 (0.51)	
Bursary	Yes	4.42 (0.65)	0.566	4.05 (1.00)	0.688	4.47 (1.23)	0.574	4.91 (0.98)	0.615	4.25 (0.87)	0.890	4.62 (0.88)	0.793	4.30 (1.13)	0.543
	No	4.38 (0.60)		4.00 (0.99)		4.38 (1.07)		4.85 (1.05)		4.24 (0.79)		4.59 (0.92)		4.21 (1.16)	
Relationship status	Single	4.39 (0.67)	0.189	4.04 (1.05)	0.951	4.34 (1.26)	0.005*	4.92 (0.96)	0.398	4.27 (0.89)	0.617	4.59 (0.91)	0.414	4.23 (1.14)	0.209
	In a relationship /married	4.46 (0.56)		4.03 (0.85)		4.74 (0.96)		4.83 (1.07)		4.22 (0.74)		4.67 (0.82)		4.40 (1.10)	
Age (in years)	18-19	4.28 (0.65)	0.043*	4.28 (0.65)	0.158	4.32 (1.25)	0.163	4.87 (0.96)	0.975	4.27 (0.81)	0.989	4.41 (0.91)	0.022*	3.91 (1.28)	<0.001*
	20-21	4.44 (0.66)		4.44 (0.66)		4.08 (1.04)		4.92 (1.04)		4.24 (0.98)		4.68 (0.87)		4.43 (1.02)	
	22-23	4.41 (0.54)		4.41 (0.54)		4.00 (0.93)		4.88 (0.95)		4.24 (0.71)		4.60 (0.85)		4.29 (1.05)	
	>23	4.60 (0.71)		4.60 (0.71)		4.30 (0.95)		4.93 (1.04)		4.28 (0.78)		4.87 (0.90)		4.63 (1.07)	
Degree	Pharmacy	4.28 (0.64)	0.016*	3.97 (1.03)	0.864	4.19 (1.24)	0.115	4.92 (0.77)	0.043*	4.17 (0.76)	0.025*	4.40 (0.85)	0.002*	4.06 (1.02)	0.240
	Optometry	4.24 (0.72)		3.94 (1.12)		4.40 (1.13)		4.77 (0.97)		4.03 (0.82)		4.26 (1.08)		4.21 (1.14)	
	Nursing	4.43 (0.62)		4.17 (1.04)		4.60 (1.23)		4.61 (1.17)		4.20 (0.80)		4.72 (0.79)		4.24 (1.20)	
	HND	4.59 (0.63)		4.01(1.02)		4.46 (1.16)		5.22 (0.81)		4.57 (1.17)		4.81 (0.87)		4.51 (1.23)	
	Medicine	4.53 (0.58)		4.08 (0.95)		4.68 (1.16)		4.97 (0.95)		4.34 (0.69)		4.80 (0.83)		4.43 (1.14)	
	Medical science	4.31 (0.67)		4.01 (0.92)		4.24 (1.19)		4.87 (1.18)		4.12 (0.85)		4.49 (0.86)		4.16 (1.03)	
Household income	R0-R5000	4.34 (0.68)	0.455	4.34 (0.68)	0.312	4.27 (1.24)	0.109	4.90 (0.95)	0.489	4.30 (1.03)	0.489	4.56 (0.98)	0.788	4.14 (1.24)	0.310
	R5001-R10000	4.48 (0.62)		4.48 (0.62)		4.71 (1.12)		4.91 (1.02)		4.27 (0.70)		4.66 (0.87)		4.23 (1.21)	
	R10001-R20000	4.46 (0.61)		4.46 (0.61)		4.43 (1.26)		4.93 (0.11)		4.31 (0.84)		4.67 (0.78)		4.34 (1.05)	
	>R20000	4.39 (0.64)		4.39 (0.64)		4.42 (1.14)		4.14 (0.76)		4.14 (0.76)		4.58 (0.90)		4.43 (1.01)	
Family residence	Rural	4.41 (0.66)	0.997	4.03 (1.01)	0.612	4.43 (1.22)	0.439	4.92 (0.96)	0.372	4.28 (0.88)	0.317	4.62 (0.89)	0.859	4.27 (1.14)	0.700
	Urban	4.41 (0.59)		4.09 (0.97)		4.54 (1.08)		4.81 (1.10)		4.17 (0.75)		4.60 (0.89)		4.32 (1.11)	
Religious affiliation	Christianity	4.43 (0.64)	0.364	4.04 (0.99)	0.898	4.46 (1.19)	0.031*	4.99 (0.94)	0.009*	4.28 (087)	0.098	4.61 (0.87)	0.958	4.27 (1.15)	0.812
	None	4.37 (0.65)		4.03 (1.03)		4.43 (1.21)		4.69 (1.07)		4.18 (0.79)		4.62 (0.92)		4.30 (1.11)	
Current living status	In university residence	4.40 (0.64)	0.905	4.04 (1.01)	0.823	4.47 (1.20)	0.811	4.87 (1.00)	0.633	4.17 (0.79)	0.650	4.62 (0.91)	0.650	4.33 (1.13)	0.180
	Private residence	4.44 (0.66)		4.06 (1.00)		4.38 (1.23)		4.98 (0.95)		4.50 (0.99)		4.55 (0.85)		4.16 (1.12)	
	Living with parents	4.40 (0.64)		3.83 (0.68)		4.56 (0.77)		5.04 (1.10)		4.31 (0.90)		4.81 (0.75)		3.73 (1.30)	
History of psychiatric illness	Yes	4.08 (0.70)	0.054	3.77 (0.86)	0.321	3.88 (1.27)	0.080	4.97 (1.12)	0.789	4.26 (0.77)	0.965	3.87 (1.15)	0.002*	3.80 (1.16)	0.120
	No	4.43 (0.64)		4.05 (1.00)		4.47 (1.19)		4.89 (0.99)		4.25 (0.85)		4.64 (0.87)		4.30 (1.13)	
Have you taken psychiatric medicine before?	Yes	4.16 (0.66)	0.128	3.71 (0.84)	0.205	4.10 (1.34)	0.130	4.71 (1.19)	0.466	4.23 (0.71)	0.918	4.18 (1.11)	0.062	4.12 (0.96)	0.595
	No	4.42 (0.64)		4.05 (1.00)		4.47 (1.19)		4.90 (0.99)		4.25 (0.85)		4.63 (0.87)		4.29 (1.14)	

***p≤ 0.05; SD: Standard Deviation; HND: Human Nutrition & Dietetics**

Discussion

It is evident from the study findings that the majority of the students that participated in this study were black, from a rural background, with low household income and stayed at the university residence. This can be attributed to the location of the university which is in the rural part of South Africa. Limpopo is one of the poorest and rural provinces of South Africa with predominantly black population. University of Limpopo was established during apartheid to provide education to blacks [22-23]. The university continues to be a first choice for the students from the disadvantaged communities in Limpopo province due to its proximity to their homes [24]

Our findings showed significant differences in the levels of the Total PMH between men and women. However, most of the students with a low level of PMH were female. These findings are in line with existing research [25-26]. For college students, stress is a major problem, and they regard their college years to be among the most stressful times of their lives. Male students have better coping with stress as they become more proactive in their stress response. It has also been demonstrated that female students experience higher levels of general and academic stress than their male counterparts [27] which could have influenced their Total PMH levels. However, other studies did not identify any total perceived stress differences in their college populations which is contrast to our study findings [28-29]. As illustrated, the existing research has produced conflicting results about the relationship between gender and perceived stress levels, which calls for further investigation. The possible explanation for significantly low levels of Total PMH in female students could also be linked to hormonal changes that occur during menstruation [30]. The above finding points to the need to put in place gender-specific interventional programmes addressing gender-specific risk factors in South African universities.

Age was found to be significantly associated with Total PMH, with students between the age of 18-19 years (first year of study) reporting significantly lower levels of PMH compared other age groups in the study in all domains excluding emotional support and interpersonal skills. Our study finding is supported by studies conducted by WHO and Dessie et al (2013) in Ethiopia that students in the first-year level of study experienced a variety of stressors than any other group of students [14, 31]. The finding may be attributed to the finding that first-year students are in their transition to university life, and the added pressure of heightened social and academic expectations puts them at risk for mental health issues. In addition, first year students' inability to navigate their way and cope with the stress of such a transition, loneliness and homesickness could have significantly influenced their PMH levels [32-33]. Results from a study conducted among a Malaysian university students revealed that factors associated with financial difficulties, demands of the university environment and the university administrative processes, and non-academic related issues were reported as underlying factors to first year students' low levels of mental well-being [34].

In this study, field of study was found to be significantly associated with total PMH, with students in HND reporting high level of Total PMH and in most domains except general coping and emotional support when compared to the students from other fields of study. Although there is no specific reason to link their significantly higher levels of Total PMH, the authors assume that their curriculum is less intense as compared to other undergraduate degrees offered by FHS.

This study identified a significant association between relationship status and emotional support. Students who were either in a relationship or married reported significantly higher scores compared to students who were single in emotional support domain. Being single was consistently linked to poor mental health over the course of a person's life, compared to marriage. Literature further suggests that compared to marriage, being single is one of the risk factors for depressive

symptoms in both men and women. Our study finding is supported by existing literature that being single is associated with poorer levels of PMH in younger people, indicating that living circumstances or legal status are not as significant at this age [35-36].

Limitations

This is a cross-sectional study conducted with comparatively a smaller group of students in FHS at University of Limpopo in Limpopo province of South Africa. Hence, the results cannot be generalised to the student population in South Africa. University of Limpopo has predominant black student population, the researchers cannot justify the effect of race on Total PMH and other domains of PMH due to the limited sample of other races. Although these limitations exist, the researchers believe that the results of this study are convincing and provide a platform for future research to further investigate the PMH levels in university students.

Conclusion

This is the first study in South Africa to measure the levels of PMH in the FHS students at a rural university in South Africa using a multi-dimensional PMH questionnaire. The study findings clearly suggest that there is a place for gender-specific interventional programmes addressing gender-specific risk factors in South African Universities. Interventions must be aimed to provide a platform for educating students regarding issues, such as mental distress, that are typically encountered during first year level of study, and on the importance of developing a range of effective social supports. Higher education institutions should make student mental health and well-being a priority issue, as it directly influences the learning process and adjustment to the academic environment. By creating a culture of well-being and providing resources and support services, universities can contribute to positive mental health outcomes for their students.

References

1. Choudhry, F.R., Mani, V., Ming, L.C. and Khan, T.M., 2016. Beliefs and perception about mental health issues: a meta-synthesis. Neuropsychiatric disease and treatment, 12, pp.2807-2818. doi: https://doi.org/10.2147/NDT.S111543
2. Van der Ham, L., Wright, P., Van, T.V., Doan, V.D. and Broerse, J.E., 2011. Perceptions of mental health and help-seeking behavior in an urban community in Vietnam: an explorative study. Community mental health journal, 47, pp.574-582. doi: https://doi.org/10.1007/s10597-011-9393-x
3. Devi, R.G., Priya, A.J. and Ranjan, M.R., 2021. Effect of Stress on Mental Health. Journal of Pharmaceutical Research International, 33, pp.491-500. doi: 10.9734/jpri/2021/v33i60A34510
4. Slade, M., 2010. Mental illness and well-being: the central importance of positive psychology and recovery approaches. BMC health services research, 10(1), pp.1-14. doi: https://doi.org/10.1186/1472-6963-10-26
5. World Health Organization, 2001. The World Health Report 2001: Mental health: new understanding, new hope. Available from: http://www.who.int/whr/2001/en/whr01_en.pdf
6. Rahmani, K., Gnoth, J. and Mather, D., 2018. Hedonic and eudaimonic well-being: A psycholinguistic view. Tourism Management, 69, pp.155-166. doi: https://doi.org/10.1016/j.tourman.2018.06.008
7. Hernández-Torrano, D., Ibrayeva, L., Sparks, J., Lim, N., Clementi, A., Almukhambetova, A., Nurtayev, Y. and Muratkyzy, A., 2020. Mental health and well-being of university students: A bibliometric mapping of the literature. Frontiers in psychology, 11, p.1226. doi: https://doi.org/10.3389/fpsyg.2020.01226
8. Rahmani, K., Gnoth, J., & Mather, D. (2018). Hedonic and eudaimonic well-being: A psycholinguistic view. Tourism Management, 69, 155-166.
9. Manchri, H., Sanagoo, A., Jouybari, L.M., Sabzi, Z. and Jafari, S.Y., 2017. The relationship between mental health status with academic performance and demographic factors among students of university of medical sciences. Journal of Nursing and Midwifery Sciences, 4(1), pp.8-13. doi: https://doi.org/10.18869/acadpub.jnms.4.1.8
10. Keyes, C.L., 2012. Promoting and protecting positive mental health: Early and often throughout the lifespan. In Mental well-being: International contributions to the study of positive mental health (pp. 3-28). Dordrecht: Springer Netherlands. doi: https://doi.org/10.1007/978-94-007-5195-8_1

11. Rady, H. and El-Sayed, L., 2015. Psychosocial and Educational Problems for Undergraduate University Nursing Students. Psychology and Behavioral Sciences, 4(3), pp.116-124. doi: 10.11648/j.pbs.20150403.15
12. Robotham, D. and Julian, C., 2006. Stress and the higher education student: a critical review of the literature. Journal of further and higher education, 30(02), pp.107-117. doi: https://doi.org/10.1080/03098770600617513
13. Hudson, D., Sacks, T., Irani, K. and Asher, A., 2020. The price of the ticket: health costs of upward mobility among African Americans. International journal of environmental research and public health, 17(4), p.1179. doi: https://doi.org/10.3390/ijerph17041179
14. Auerbach, R.P., Mortier, P., Bruffaerts, R., Alonso, J., Benjet, C., Cuijpers, P., Demyttenaere, K., Ebert, D.D., Green, J.G., Hasking, P. and Murray, E., 2018. WHO world mental health surveys international college student project: prevalence and distribution of mental disorders. Journal of abnormal psychology, 127(7), p.623. doi: https://doi.org/10.1037/abn0000362
15. Pandya, A. and Lodha, P., 2022. Mental health consequences of COVID-19 pandemic among college students and coping approaches adapted by higher education institutions: A scoping review. SSM-Mental Health, 2, p.100122. doi: https://doi.org/10.1016/j.ssmmh.2022.100122
16. Pei Boon, O., Khor, K. S., Tan, C. C., & DLT, O. (2022). Depression, anxiety, stress and satisfaction with life: moderating role of interpersonal needs amongst university students. Frontiers in Public Health. doi: https://doi.org/10.3389/fpubh.2022.958884
17. Siegel, D.M., Burke, T.A., Hamilton, J.L., Piccirillo, M.L., Scharff, A. and Alloy, L.B., 2018. Social anxiety and interpersonal stress generation: the moderating role of interpersonal distress. Anxiety, Stress, & Coping, 31(5), pp.526-538. doi: 10.1080/10615806.2018.1482723
18. Garagiola, E.R., Lam, Q., Wachsmuth, L.S., Tan, T.Y., Ghali, S., Asafo, S. and Swarna, M., 2022. Adolescent resilience during the COVID-19 pandemic: a review of the impact of the pandemic on developmental milestones. Behavioral Sciences, 12(7), p.220. doi: https://doi.org/10.3390/bs12070220
19. Gao, H., 2022. Research on the construction of college students' mental health security system. Journal of healthcare engineering, 2022. doi: https://doi.org/10.1155/2022/4001603
20. Deasy, C., Coughlan, B., Pironom, J., Jourdan, D. and Mannix-McNamara, P., 2014. Psychological distress and coping amongst higher education students: A mixed method enquiry. Plos one, 9(12), p.e115193. doi: https://doi.org/10.1371/journal.pone.0115193

21. Vaingankar, J.A., Subramaniam, M., Chong, S.A., Abdin, E., Orlando Edelen, M., Picco, L., Lim, Y.W., Phua, M.Y., Chua, B.Y., Tee, J.Y. and Sherbourne, C., 2011. The positive mental health instrument: development and validation of a culturally relevant scale in a multi-ethnic Asian population. Health and quality of life outcomes, 9, pp.1-18. doi: https://doi.org/10.1186/1477-7525-9-92
22. Seroto, J., 2011. The provision of rural education in three provinces of South Africa since 1994: Implications for school improvement. Journal of Educational Studies, 2011(si-1), pp.138-152. Available from: https://hdl.handle.net/10520/EJC160134
23. d Zeelen, J., Rampedi, M. and van der Linden, J., 2014. Grounding adult education research in rural areas: Reflections on the development of a research program at the University of Limpopo in South Africa. Adult Education Quarterly, 64(1), pp.20-38. doi: https://doi.org/10.1177/0741713613495086
24. Mattern, K. and Wyatt, J.N., 2009. Student Choice of College: How Far Do Students Go for an Education?. Journal of College Admission, 203, pp.18-29.
25. Sequeira, C., Carvalho, J.C., Gonçalves, A., Nogueira, M.J., Lluch-Canut, T. and Roldán-Merino, J., 2020. Levels of positive mental health in Portuguese and Spanish nursing students. Journal of the American Psychiatric Nurses Association, 26(5), pp.483-492. doi: https://doi.org/10.1177/1078390319851569
26. Matud, M.P., López-Curbelo, M. and Fortes, D., 2019. Gender and psychological well-being. International journal of environmental research and public health, 16(19), p.3531. doi: https://doi.org/10.3390/ijerph16193531
27. Rahardjo, W., Juneman, J. and Setiani, Y., 2013. Computer anxiety, academic stress, and academic procrastination on college students. Journal of Education and Learning (EduLearn), 7(3), pp.147-152. doi: https://doi.org/10.11591/edulearn.v7i3.179
28. Jones, D.R., Lehman, B.J., Noriega, A. and Dinnel, D.L., 2019. The effects of a short-term mindfulness meditation intervention on coping flexibility. Anxiety, Stress, & Coping, 32(4), pp.347-361. doi: https://doi.org/10.1080/10615806.2019.1596672
29. Deckro, G.R., Ballinger, K.M., Hoyt, M., Wilcher, M., Dusek, J., Myers, P., Greenberg, B., Rosenthal, D.S. and Benson, H., 2002. The evaluation of a mind/body intervention to reduce psychological distress and perceived stress in college students. Journal of American College Health, 50(6), pp.281-287. doi: https://doi.org/10.1080/07448480209603446

30. Stallman, H.M., 2010. Psychological distress in university students: A comparison with general population data. Australian psychologist, 45(4), pp.249-257. doi: https://doi.org/10.1080/00050067.2010.482109

31. Dessie, Y., Ebrahim, J. and Awoke, T., 2013. Mental distress among university students in Ethiopia: a cross sectional survey. Pan African Medical Journal, 15(1). doi: 10.11604/pamj.2013.15.95.2173

32. Mutinta, G., 2022. Mental distress among university students in the Eastern Cape Province, South Africa. BMC psychology, 10(1), p.204. doi: https://doi.org/10.1186/s40359-022-00903-8

33. Modipane, M.C., 2011. Initial experiences of first entering students at the University of Limpopo: Implications for coping with academic work/studies. South African Journal of Higher Education, 25(8), pp.1592-1607. Available from: https://hdl.handle.net/10520/EJC121489

34. Shamsuddin, K., Fadzil, F., Ismail, W.S.W., Shah, S.A., Omar, K., Muhammad, N.A., Jaffar, A., Ismail, A. and Mahadevan, R., 2013. Correlates of depression, anxiety and stress among Malaysian university students. Asian journal of psychiatry, 6(4), pp.318-323. doi: https://doi.org/10.1016/j.ajp.2013.01.014

35. Simon, R.W. and Barrett, A.E., 2010. Nonmarital romantic relationships and mental health in early adulthood: Does the association differ for women and men?. Journal of Health and Social Behavior, 51(2), pp.168-182. doi: https://doi.org/10.1177/0022146510372343

36. Uecker, J.E., 2012. Marriage and mental health among young adults. Journal of Health and Social Behavior, 53(1), pp.67-83. doi: https://doi.org/10.1177/0022146511419206
